# *Epicoccum* Associated with Soybean in Russia: Species Diversity, Pathogenic Potential, and Genomic Features

**DOI:** 10.3390/plants15213388

**Published:** 2026-11-01

**Authors:** Viktoriya V. Medvedeva, Nikita K. Kostin, Kirill M. Vorobev, Rashit I. Tarakanov, Peter V. Evseev, Anna A. Vasilyeva, Olga Yu. Slovareva, Svetlana I. Chebanenko, Olga O. Beloshapkina, Fevzi S.-U. Dzhalilov

**Affiliations:** 1 Department of Plant Protection, Russian State Agrarian University-Moscow Timiryazev Agricultural Academy, Timiryazevskaya Str. 49, 127434 Moscow, Russia; vikamedm@mail.ru (V.V.M.); kostinwork1@gmail.com (N.K.K.); kirillvrbv@mail.ru (K.M.V.); annavasilyeva.bioscience@gmail.com (A.A.V.); slovareva.olga@gmail.com (O.Y.S.); svchebanenko@rgau-msha.ru (S.I.C.); beloshapkina@rgau-msha.ru (O.O.B.); dzhalilov@rgau-msha.ru (F.S.-U.D.); 2 All-Russian Plant Quarantine Center, Pogranichnaya Str. 32, 140150 Moscow, Russia; 3 Laboratory of Molecular Microbiology, Pirogov Russian National Research Medical University, Ostrovityanova 1, 117997 Moscow, Russia; petevseev@gmail.com

**Keywords:** *Epicoccum*, *Glycine max*, multilocus phylogeny, comparative genomics, pathogenic potential, seed colonization, seed germination, extracellular enzymes, fungicide sensitivity

## Abstract

Species of the genus *Epicoccum* are widespread plant-associated fungi; however, their phytopathological significance in soybean remains insufficiently studied. In the present study, 15 *Epicoccum* isolates obtained in 2024–2026 from symptomatic soybean leaves and seeds in four regions of Russia were characterized using multilocus phylogenetic analysis, whole-genome sequencing, controlled inoculation assays, assessment of extracellular enzymatic activity, and fungicide sensitivity testing. Nine isolates were identified as *E. tobaicum*, five as *E. mezzettii*, and one as *E. oryzae*. The five sequenced genomes ranged from 32.84 to 34.62 Mb in size, with BUSCO completeness of 97.1–98.9%. In the mycelial-plug assay, performed without needle puncturing of the leaf blade, the largest mean necrotic lesion diameter was 12.75 ± 5.03 mm; in a separate wound-assisted assay, in which an *E. tobaicum* conidial suspension was applied to standardized needle wounds, the highest mean proportion of necrotic leaflet area was 26.17 ± 8.93% at 7 days after inoculation. Following artificial seed inoculation with *E. tobaicum* isolate 152H, no normal seedlings developed, and visible signs of fungal colonization were recorded on 100% of the seeds, indicating substantial adverse effects on seedling development under the experimental conditions. For 14 of the 15 isolates, 50% inhibition of mycelial growth by azoxystrobin was not reached at the highest tested concentration (EC_50_ > 1000 µg/mL), indicating low sensitivity under the test conditions, whereas EC_50_ values ranged from 0.0448 to 0.3837 µg/mL for boscalid, from 0.0760 to 0.2736 µg/mL for fludioxonil, and from 3.735 to 49.542 µg/mL for tebuconazole. These findings demonstrate substantial variation in fungicide sensitivity among the tested isolates, although further comparative studies are required to establish resistance status. To our knowledge, this study reports the first molecularly confirmed detection of *E. tobaicum*, *E. mezzettii*, and *E. oryzae* associated with soybean in Russia, as well as the first experimental evidence of the pathogenic potential of *E. tobaicum* toward soybean leaves in the country under controlled inoculation conditions. Because the conidial assay was performed on wounded leaflets, it does not establish the ability of these isolates to infect intact leaves under natural conditions.

## 1. Introduction

Soybean (*Glycine max* (L.) Merr.) is one of the world’s most important grain legume and oilseed crops and is becoming increasingly important in Russian crop production. According to the latest available FAOSTAT data, in 2024 the soybean cultivation area in the Russian Federation was approximately 4.13 million ha, and production reached 7.00 million t [1]. Over the past decade, the cultivated area and total production of this crop in the country have increased substantially, emphasizing the importance of phytosanitary monitoring and investigation of the complex of microorganisms associated with soybean. Recent studies of Russian agroecosystems have revealed a diverse soybean mycobiota comprising both well-known phytopathogenic fungi and less-studied taxa whose roles in disease development remain unclear [2]. In this context, particular interest lies in fungi capable of occupying different ecological niches and exhibiting saprotrophic, endophytic, antagonistic, or phytopathogenic lifestyles.

Such microorganisms include members of the genus *Epicoccum* Link (*Didymellaceae*, *Pleosporales*), which are widespread in soil, air, living and dead plant tissues, seeds, and stored agricultural products. The taxonomy of the genus has been substantially revised in recent years. Many isolates previously identified as *E. nigrum* or assigned to the morphologically broad *Phoma* complex have been separated into distinct species on the basis of multilocus phylogenetic analysis [3,4]. Consequently, accurate assessment of the host range, pathogenicity, and other biological properties of *Epicoccum* species requires species-level identification using molecular markers.

Although *Epicoccum* species have traditionally been regarded primarily as saprotrophic and endophytic fungi, the number of species with confirmed phytopathogenic activity continues to increase. Information on 18 *Epicoccum* species associated with diseases of 46 plant species in 20 countries has been summarized, with leaf spot being the most common symptom [5]. Members of the genus have been reported in cereal, forage, grain legume, vegetable, industrial, fruit, and ornamental crops. One of the most widely distributed species is *E. sorghinum*, which has been reported from maize, sorghum, rice, sugarcane, bean, tea, tobacco, and other plants [5,6]. Diseases associated with *Epicoccum* species may manifest as leaf spots and blights, lesions of stems and leaf sheaths, grain and seed discoloration, reduced seed germination, and postharvest disorders.

The pathogenic potential of *Epicoccum* varies substantially among species and individual isolates. *Epicoccum nigrum*, *E. layuense* (currently treated as a synonym of *E. tobaicum*), *E. sorghinum*, *E. latusicollum*, and *E. pneumoniae* were identified from diseased maize leaves in China, and the pathogenicity of all five species was experimentally confirmed [6]. The isolates also differed in aggressiveness, extracellular enzyme production, and fungicide sensitivity. In a study of leaf spot on Italian ryegrass (*Lolium multiflorum*) in southwestern China, 202 *Epicoccum* isolates representing ten species were obtained. Six species, including *E. tobaicum* and *E. oryzae*, were pathogenic, and disease severity differed substantially among species and isolates [7]. In contrast, *E. mezzettii* is much more frequently described as a plant-associated or antagonistic fungus, including as a potential biological control agent, whereas information on its pathogenicity remains limited [5].

The association of *Epicoccum* species with soybean has been studied much less extensively. For a long time, fungi of this genus were considered primarily components of the mycobiota associated with soybean seeds and other organs, without experimental confirmation of their etiological role. For example, in Poland, *E. nigrum* accounted for 3.9% of all fungal isolates obtained from stored soybean seeds [8]. In western regions of Ukraine, the mean frequency of *Epicoccum* spp. detection on soybean seeds was 2.3%, reaching 12.3% in individual samples [9]. These studies confirm the regular presence of members of the genus in the soybean seed mycobiota; however, detection alone does not establish pathogenicity.

Direct experimental evidence that *Epicoccum* can cause soybean diseases has emerged only in recent years. *Epicoccum sorghinum* was established as the causal agent of soybean leaf spot in Heilongjiang Province, China [10]. Artificial inoculation produced characteristic dark-brown necrotic lesions with chlorosis of the surrounding tissues, and the fungus was reisolated from the resulting symptoms. Subsequently, *E. nigrum* was identified as the causal agent of soybean leaf blight in northeastern China; the disease was characterized by enlarging brown necrotic lesions, often delimited by veins and surrounded by a yellow peripheral zone [11]. The pathogenic potential of the genus also extends to seed material. The ability of *E. italicum* to cause soybean seed disease in North Dakota, USA, has been experimentally confirmed [12]. Inoculation resulted in brown-to-black discoloration and the development of superficial mycelium, while seed germination decreased from 100% in the control to 70% in the inoculated treatment. Experimental confirmation of pathogenicity on soybean has therefore been obtained for *E. sorghinum*, *E. nigrum*, and *E. italicum* [10,11,12]. However, the pathogenic potential of *E. tobaicum*, *E. oryzae*, and especially *E. mezzettii* on soybean remains largely unexplored, despite the reported pathogenicity of the first two species on other plant hosts [6,7].

One characteristic feature of *Epicoccum* species is their pronounced phenotypic heterogeneity. Even isolates belonging to the same species may differ substantially in aggressiveness, growth rate, and the ability to produce extracellular hydrolytic enzymes. Production of pectinases, cellulases, amylases, proteases, lipases, and other enzymes potentially involved in colonization and degradation of plant tissues has been described for members of the genus. Significant differences in the production of pectinase, cellulase, protease, amylase, laccase, and gelatinase have been detected among *Epicoccum* isolates obtained from maize [6]. However, the relationship between enzymatic activity and aggressiveness in soybean-associated isolates has scarcely been investigated. Comparative genomic data for soybean-associated members of the genus have also been limited, including information on secreted proteins, carbohydrate-active enzymes, potential effectors, and other functional repertoires that may be associated with fungus-plant interactions.

The fungicide sensitivity of phytopathogenic *Epicoccum* species is also insufficiently studied. A substantial proportion of earlier studies focused on the compatibility of antagonistic isolates with chemical plant protection products, whereas quantitative data for pathogenic populations remain fragmentary. Pronounced variability in sensitivity to carbendazim and tebuconazole has been demonstrated among maize-derived isolates [6]. The sensitivity of *E. nigrum* causing soybean leaf blight was subsequently investigated, revealing high activity of individual fungicides against this pathogen [11]. However, quantitative data on the sensitivity of *E. tobaicum*, *E. mezzettii*, and *E. oryzae* to fungicides with different modes of action remain extremely limited.

Information on the diversity and phytopathological significance of *Epicoccum* species in Russia is also fragmentary. Studies of the family *Didymellaceae* have confirmed the presence of diverse members of the genus in the country and have led, in particular, to the description of *E. convolvulicola* and *E. pseudoplurivorum* [13]. Members of *Epicoccum*, including *E. nigrum*, have been detected in the soybean mycobiota of Russian agroecosystems; however, detection of a fungus in plant material is not equivalent to evidence of its etiological role [2]. To our knowledge, *E. tobaicum*, *E. mezzettii*, and *E. oryzae* have not previously been molecularly confirmed on soybean in Russia, and their pathogenicity toward this crop has not been experimentally investigated in the country.

The aim of the present study was to comprehensively characterize *Epicoccum* isolates obtained from soybean leaves and seeds in Russia. Species identity was determined using multilocus phylogenetic analysis, and comparative whole-genome analysis was performed for five deliberately selected isolates. Cultural and morphological characteristics, leaf responses and isolate aggressiveness under controlled inoculation conditions, effects on seed germination and seedling development, extracellular enzymatic activity, and in vitro sensitivity to azoxystrobin, boscalid, fludioxonil, and tebuconazole were also assessed. This integrated approach enabled the characterization of the taxonomic and functional diversity of soybean-associated *Epicoccum* and an evaluation of their potential phytopathological significance without assuming an etiological role from isolation alone.

## 2. Results

### 2.1. Phytosanitary Monitoring, Symptoms of the Original Samples, and Composition of the Epicoccum Isolate Collection

During phytosanitary monitoring aimed at studying the fungal disease complex of soybean, leaves and seeds showing visible signs of damage of putative fungal origin were collected. The monitoring was not designed to specifically target members of the genus *Epicoccum*; therefore, the observed symptoms were used exclusively as criteria for selecting material for subsequent mycological analysis and were not considered diagnostic features of this genus.

**Figure 1:**
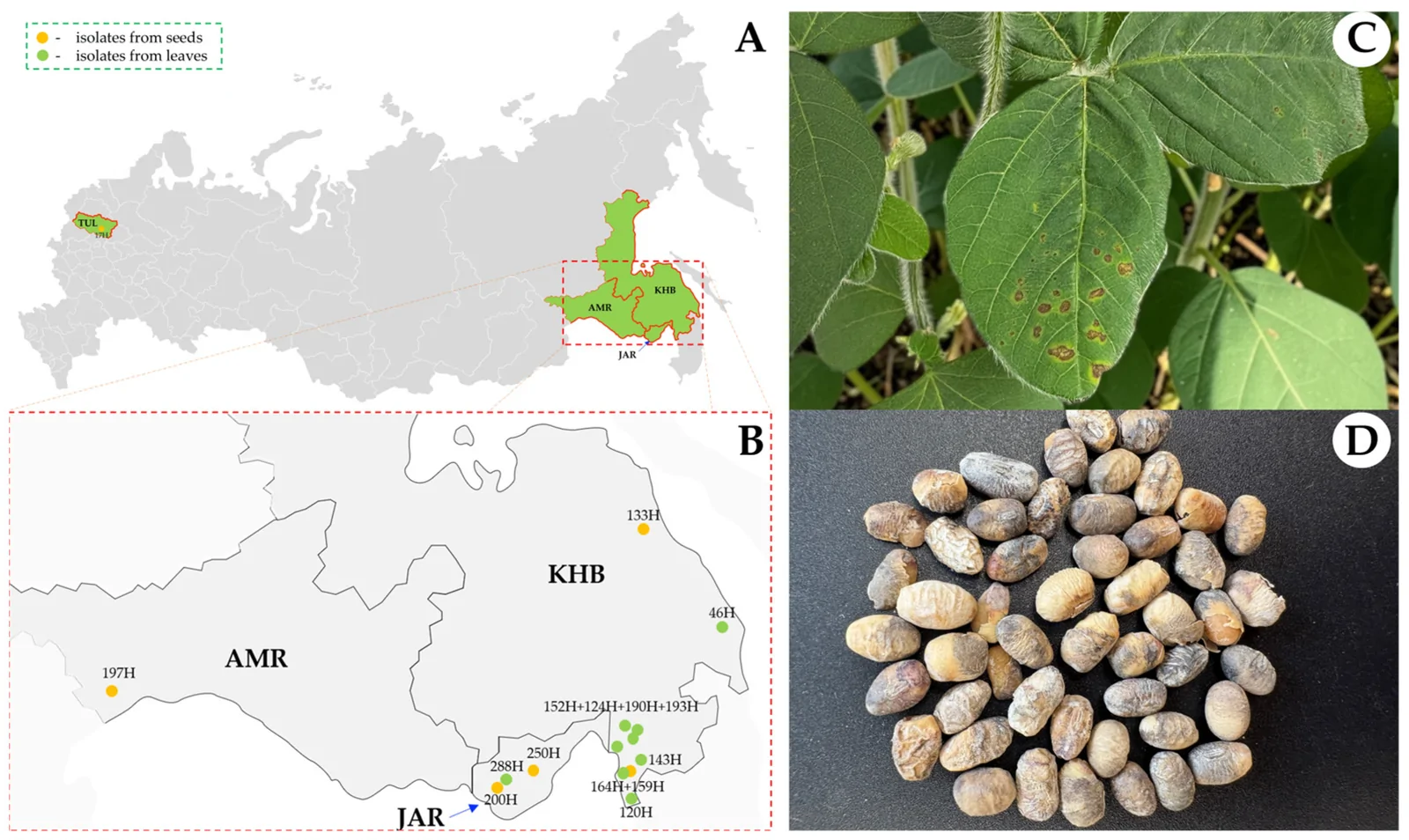
Geographic origin of the studied *Epicoccum* isolates and external signs of damage in the original soybean samples collected during phytosanitary monitoring of the fungal disease complex. (**A**) Locations of the surveyed regions of Russia; (**B**) detailed locations of sampling sites in the Russian Far East; (**C**) necrotic leaf lesions used as one of the criteria for material selection; (**D**) spotting and discoloration of the seed surface. Point codes correspond to isolate IDs in Table 1. Orange points indicate isolates obtained from seeds, and green points indicate isolates obtained from leaves. TUL—Tula region; AMR—Amur region; KHB—Khabarovsk region; JAR—Jewish Autonomous region.

Single and multiple necrotic spots of circular or irregular shape, predominantly light brown, brown, and dark brown, were observed on leaves of the surveyed plants. In some lesions, the central area was lighter and the periphery darker; closely spaced spots could coalesce to form large necrotic areas. The seeds exhibited uneven spotting and seed-coat discoloration, with yellowish-brown, grayish-brown, and dark-brown areas of varying size, as well as localized surface darkening (Figure 1). The observed symptoms were not specific to *Epicoccum*, could be associated with different components of the soybean fungal disease complex, and were therefore insufficient to establish the identity of the causal fungi. Accordingly, the identification of the *Epicoccum* isolates relied on culture isolation followed by morphological characterization and multilocus molecular analysis.

Mycological analysis of the collected material yielded a study collection of 15 *Epicoccum* isolates, comprising 10 isolates from soybean leaves and five from soybean seeds. Of these, 10 isolates originated from the Khabarovsk region, three from the Jewish Autonomous region, and one each from the Amur region and the Tula region. The geographic locations of the sampling sites and isolation sources are shown in Figure 1, and detailed information for each isolate is provided in Table 1. Nine isolates were collected in 2024, five in 2025, and one in 2026. By species, *E. tobaicum* comprised seven leaf isolates and two seed isolates, *E. mezzettii* three leaf isolates and two seed isolates, and *E. oryzae* a single seed isolate. Because the sampling was symptom-directed and was not designed as a prevalence survey, these counts should not be interpreted as estimates of the regional frequency, prevalence, or population structure of *Epicoccum* associated with soybean in Russia, and the collection may not represent the full diversity of the genus on this crop in the country.

Subsequent multilocus identification based on ITS, *RPB2*, and *TUB2* sequences confirmed that all 15 isolates belonged to the genus *Epicoccum* and assigned them to three species: nine isolates to *E. tobaicum*, five to *E. mezzettii*, and one to *E. oryzae*. Representatives of *E. tobaicum* and *E. mezzettii* were obtained from both leaves and seeds, whereas the single *E. oryzae* isolate, 164H, was isolated from soybean seeds in the Khabarovsk region. Species identity, isolate origin, and GenBank accession numbers for ITS, *RPB2*, and *TUB2* are provided in Table 1.

All 15 isolates were used for subsequent cultural-morphological and phenotypic characterization. Five isolates were deliberately selected for comparative whole-genome analysis so that the set represented all three phylogenetic lineages identified in the multilocus analysis: *E. mezzettii* 46H, *E. oryzae* 164H, and *E. tobaicum* 152H, 193H, and 288H. Within the most numerous *E. tobaicum* lineage, the three isolates were chosen to include contrasting phenotypic profiles observed in the present study. This five-genome set was intended for exploratory comparative analysis and was not treated as a population sample.

### 2.2. Multilocus Identification and Comparative Genomics of the Isolates

#### 2.2.1. Multilocus Phylogenetic Identification

**Figure 2:**
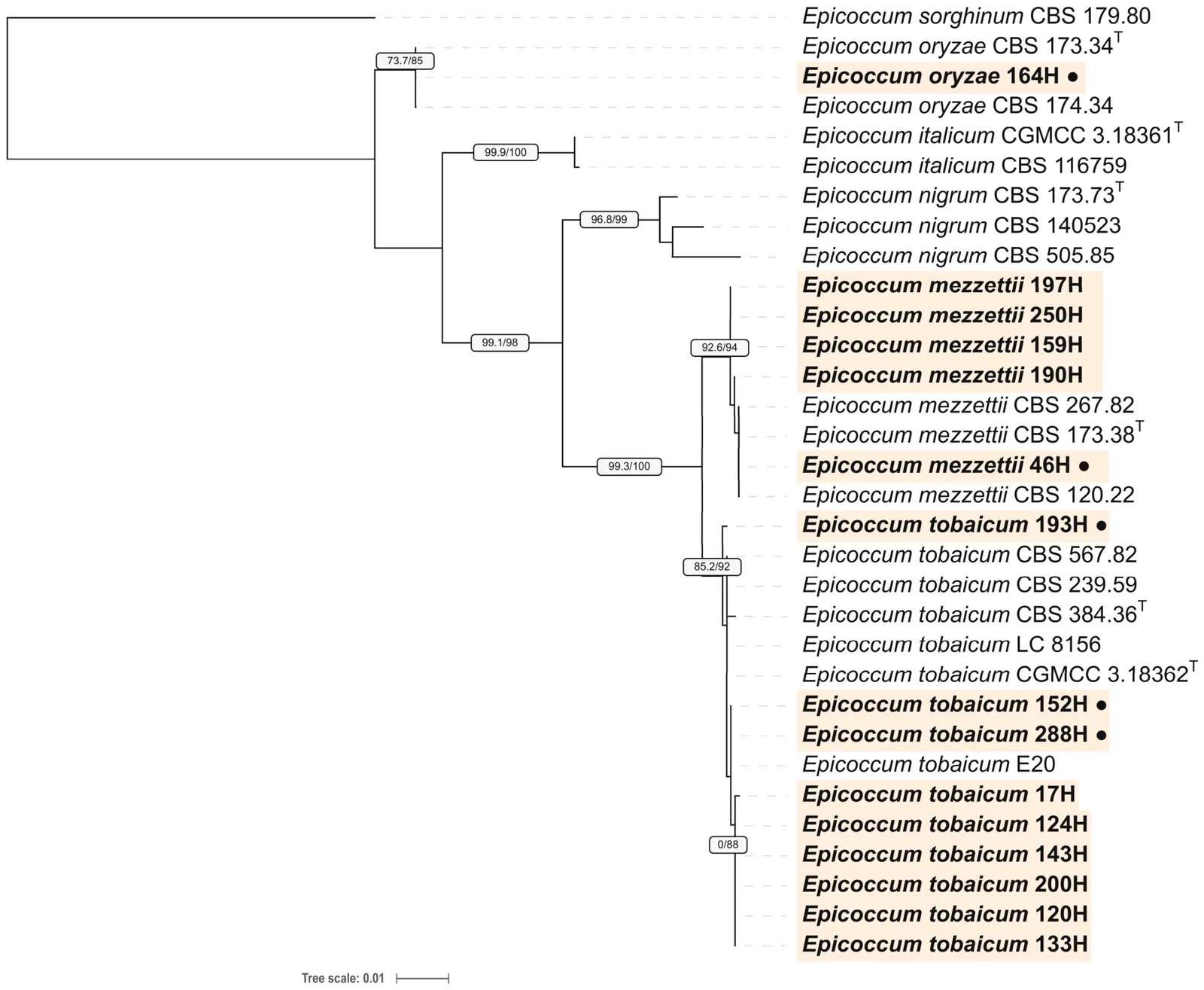
Phylogenetic position of the studied *Epicoccum* isolates based on concatenated ITS, *RPB2*, and *TUB2* sequences. The tree was inferred using the maximum-likelihood method; node support values are shown as SH-aLRT/UFBoot (%). *Epicoccum sorghinum* CBS 179.80 was used as the outgroup. Isolates obtained in the present study are shown in bold and highlighted; isolates selected for whole-genome sequencing are indicated by the symbol ●. Type and ex-type cultures are indicated by a superscript T.

The phylogenetic tree based on the concatenated ITS, *RPB2*, and *TUB2* dataset resolved the 15 study isolates into three lineages corresponding to *E. tobaicum*, *E. mezzettii*, and *E. oryzae* (Figure 2). Nine isolates (17H, 120H, 124H, 133H, 143H, 152H, 193H, 200H, and 288H) clustered with *E. tobaicum* (SH-aLRT/UFBoot = 85.2/92), five isolates (46H, 159H, 190H, 197H, and 250H) clustered with *E. mezzettii* (92.6/94), and 164H was positioned within the broader *E. oryzae* lineage (73.7/85). The placement of 164H relative to CBS 174.34 was not sufficiently resolved; therefore, 164H was assigned to the broader E. oryzae lineage rather than interpreted as closely related to a particular reference strain.

Identical concatenated profiles were detected for the isolate groups 120H/124H/133H/143H/200H, 159H/197H/250H, and 152H/288H. None of the study isolates clustered with the reference lineages of *E. nigrum* or *E. italicum*. Among the five isolates selected for whole-genome sequencing, 152H, 193H, and 288H were assigned to *E. tobaicum*, 46H to *E. mezzettii*, and 164H to the *E. oryzae* lineage.

#### 2.2.2. Whole-Genome Characterization and Comparative Genomic Analysis

The five final genome assemblies ranged from 32.84 to 34.62 Mb in size, with 1030–1780 contigs, N50 values of 57,075–117,457 bp, GC contents of 52.24–52.94%, and mean raw-read coverage depths of 242.61-357.51×. The proportion of mapped reads was 96.77–98.36%, and 98.95–99.80% of the final assembly lengths had coverage of at least 20×. The annotations predicted 10,814–11,573 protein-coding genes and 174–192 tRNAs. The proportions of complete BUSCOs were 97.1–98.9% for the genome assemblies and 95.1–97.0% for the proteomes. Assembly 288H was the most fragmented (1780 contigs; N50 = 57,075 bp) but retained 97.6% complete BUSCOs and 98.95% of the assembly length at coverage of at least 20×. Full quality metrics and deposition identifiers are provided in Supplementary Table S2.

The highest FungANI values were observed among the three *E. tobaicum* genomes: 98.8795% for 152H–288H, 97.6598% for 152H–193H, and 97.6578% for 193H–288H. FungANI values between *E. mezzettii* 46H and the three *E. tobaicum* genomes ranged from 94.1012 to 94.1620%, whereas comparisons involving *E. oryzae* 164H yielded values ranging from 89.5932 to 89.7712%. The complete symmetric FungANI matrix is provided in Supplementary Table S3.

The predicted functional repertoires were broadly similar in size across the five genomes. The predicted consensus classical secretomes comprised 802–867 proteins, of which 220–252 were classified by EffectorP as candidate effectors. The number of high-confidence CAZymes was 545–557, including 285–292 secreted proteins. The number of proteins with filtered PHI-base homologs was 2850–2928. antiSMASH identified 35–43 predicted BGC regions per genome; because the assemblies were fragmented, these values do not necessarily correspond to the numbers of complete independent biosynthetic gene clusters. These secretome, candidate effector, CAZyme, PHI-base, and BGC assignments are based on computational prediction and/or sequence homology; their involvement in soybean infection was not experimentally validated. Detailed values are provided in Supplementary Table S3.

#### 2.2.3. Orthogroup Analysis and Candidate Genomic Differences

OrthoFinder assigned 54,911 of 56,081 proteins (97.9%) to 11,393 orthogroups; 9243 orthogroups were present in all five genomes, and 8682 of these were single-copy orthogroups. Only 16 orthogroups, comprising a total of 35 genes, were restricted to one of the five genomes examined. These orthogroups were considered isolate-associated within this five-genome comparison rather than species-specific.

The most pronounced validated copy-number difference was detected for OG0000216, representing a spherulin/germin-like subfamily of proteins, which contain the Cupin_1 domain. Four predicted protein models in 152H belonged to OG0000216, whereas 164H, 193H, and 288H each contained one; no protein models from this specific orthogroup were detected in the 46H proteome. This difference concerns the composition of a specific orthogroup and does not imply the absence of cupin-like sequences as a class from the other genomes. The four 152H paralogs formed a monophyletic group; OrthoFinder identified three terminal duplication events on the 152H lineage with duplication-support = 1.0. All four loci were located on different contigs and had 100% raw-read coverage; after filtering reads at MAPQ ≥ 20, 95.9–98.0% of the local coverage depth was retained. Homologous blocks approximately 3.1–5.0 kb in length with 90.3–94.7% nucleotide identity were detected between flanking regions of individual loci, which is consistent with the copies originating within larger duplicated genomic segments but does not by itself establish the exact structural mechanism of duplication. Detailed validation of OG0000216, as well as supported, deprioritized, and excluded candidate differences after verification, is provided in Supplementary Table S4. The co-occurrence of the expanded OG0000216 orthogroup and the pronounced phenotype of isolate 152H is descriptive. Because no gene-disruption, expression, or complementation experiments were performed, no causal relationship between this copy-number pattern and aggressiveness or seed effects can be inferred from the present data.

### 2.3. Cultural-Morphological Characteristics and Growth of the Isolates

#### 2.3.1. Cultural Morphology and Sporulation

**Figure 3:**
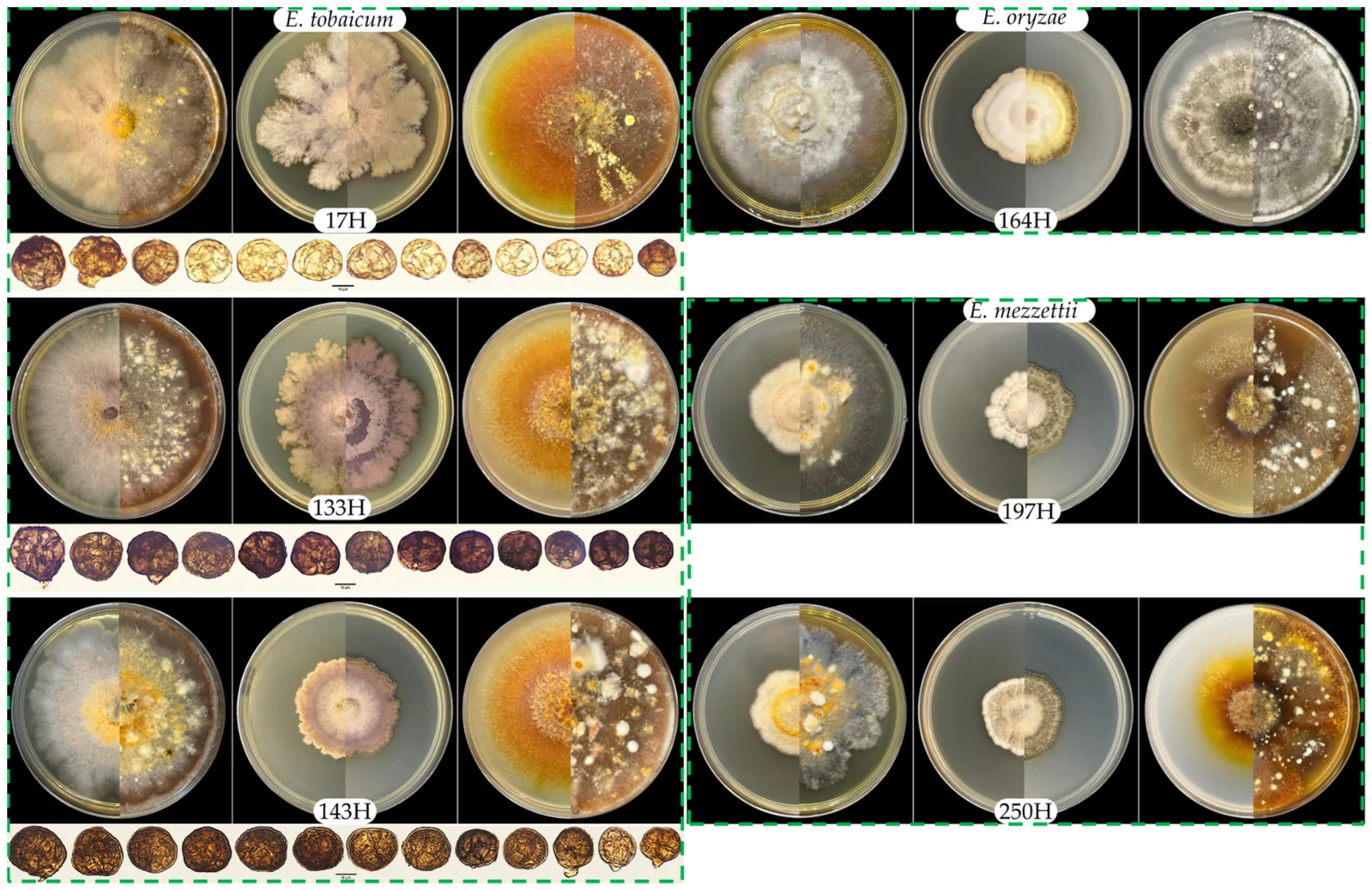
Cultural morphology and conidia of representative *Epicoccum* isolates obtained from soybean. Isolates *E. tobaicum* 17H, 133H, and 143H, *E. oryzae* 164H, and *E. mezzettii* 197H and 250H are shown. For each isolate, colonies on potato dextrose agar (PDA), malt extract agar (MEA), and oatmeal agar (OA) are presented. Each circular image combines photographs of the colony after 10 days (left half) and 20 days (right half) of cultivation at 25 °C. Micrographs of conidia formed on PDA are shown beneath the colony images of *E. tobaicum*. Conidia of *E. mezzettii* and *E. oryzae* were not obtained under the cultivation conditions used. Scale bars = 10 µm.

On PDA, MEA, and OA, the studied *Epicoccum* isolates differed in colony color and structure, development of aerial mycelium, margin characteristics, zonation, and pigmentation. The most pronounced cultural variability was observed among *E. tobaicum* isolates: colonies ranged from whitish-gray and cream to ochre, orange-brown, and dark brown, often with pronounced zonation and heterogeneous aerial mycelium. Particularly intense colony darkening was observed for isolate 152H, whereas 288H was characterized by a more irregular, lobate margin. On OA, most *E. tobaicum* isolates exhibited pronounced yellow-orange or ochre pigmentation (Figure 3).

*Epicoccum mezzettii* isolates 46H, 159H, 190H, 197H, and 250H predominantly formed more compact cream, gray, or gray-olive colonies; the most pronounced darkening and ochre-brown pigmentation developed on OA. The single *E. oryzae* isolate examined, 164H, was characterized by relatively light whitish-gray colonies with pronounced aerial mycelium and concentric zonation. Because only one *E. oryzae* isolate was included in the study, these features were regarded as characteristics of isolate 164H rather than diagnostic features of the species.

Sporulation on PDA was obtained for all nine *E. tobaicum* isolates examined: 17H, 120H, 124H, 133H, 143H, 152H, 193H, 200H, and 288H. Conidia were predominantly globose or subglobose, multicellular, yellow-brown to dark brown, with distinct transverse and longitudinal septa and an uneven surface. Their dimensions were approximately 15.6-31.4 × 13.7–25.5 µm, with a mean of 23.4 ± 3.0 × 20.9 ± 2.4 µm. Conidia could not be obtained from the *E. mezzettii* and *E. oryzae* isolates examined under the cultivation conditions used (Figure 3).

#### 2.3.2. Colony Growth Rate on Different Culture Media

**Figure 4:**
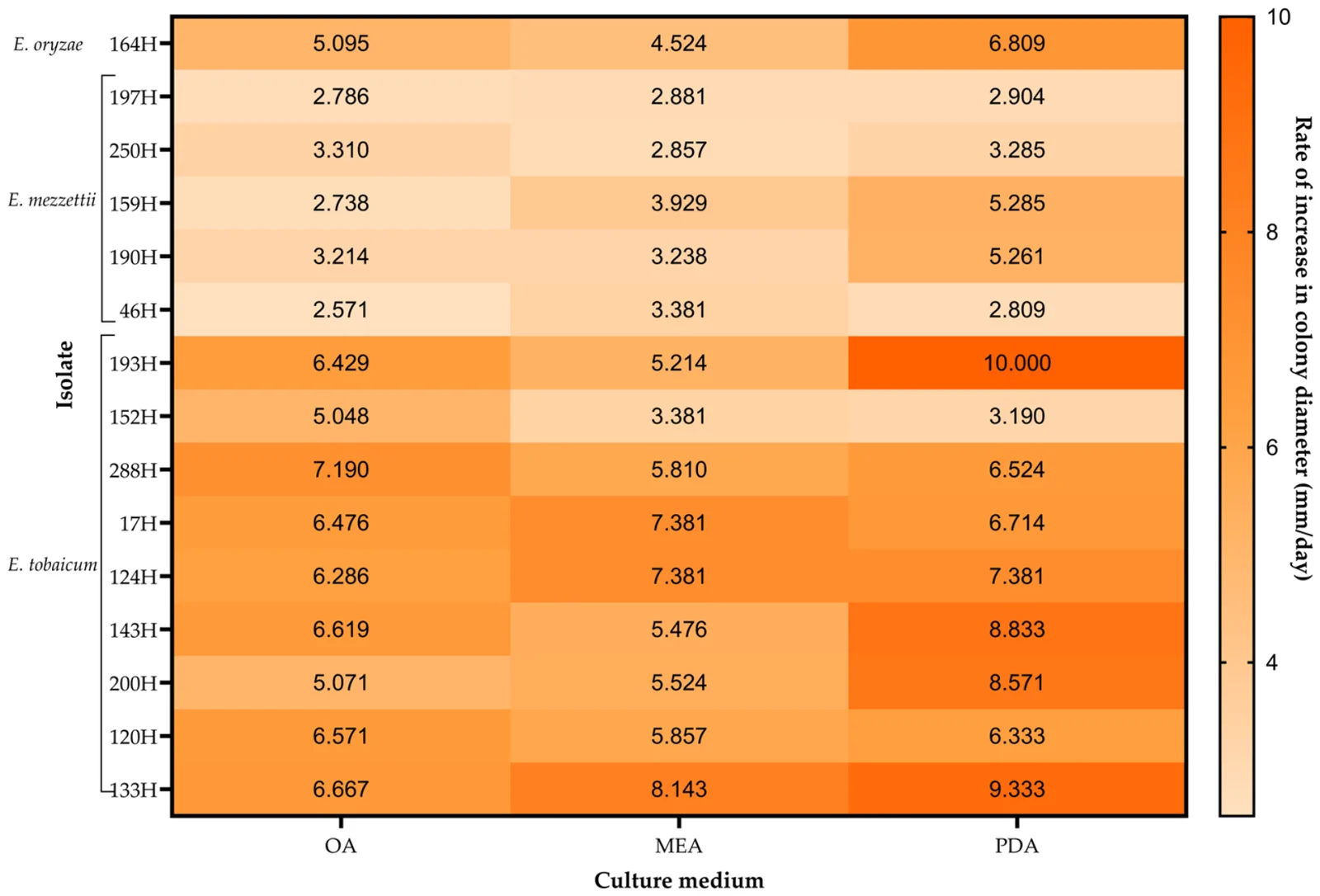
Mean rate of increase in colony diameter of the studied *Epicoccum* isolates on oatmeal agar (OA), malt extract agar (MEA), and potato dextrose agar (PDA). Heatmap cells show mean values in mm/day; color intensity corresponds to the magnitude of the parameter. Complete mean ± standard deviation values and results of Duncan’s multiple range test are provided in Table S5.

The rate of increase in colony diameter differed among isolates on PDA, MEA, and OA (one-way ANOVA performed separately for each medium; *p* < 0.001 in all cases; Figure 4; Table S13). On PDA, values ranged from 2.810 ± 0.541 mm/day for isolate 46H to 10.000 ± 0.143 mm/day for 193H. Isolate 193H had the highest growth rate and belonged to statistical group a; high values were also recorded for 133H (9.333 ± 0.297 mm/day; ab) and 143H (8.833 ± 0.352 mm/day; bc). The lowest values on PDA were recorded for 46H, 197H, 250H, and 152H, which did not differ from one another and belonged to group g.

On MEA, growth rates ranged from 2.857 ± 0.143 mm/day for 250H to 8.143 ± 0.286 mm/day for 133H. Isolate 133H formed the fastest-growing colonies and constituted a separate statistical group, a, whereas the lowest values were observed for 197H and 250H (group h). On OA, the lowest growth rate was recorded for 46H (2.571 ± 0.286 mm/day) and the highest for 288H (7.190 ± 0.165 mm/day), which constituted group a. Based on mean values, isolate ranking differed among media: the highest growth rate was recorded for 193H on PDA, 133H on MEA, and 288H on OA. These descriptive differences are not the result of a separate statistical test of the medium effect or the isolate × medium interaction. Complete values and results of multiple comparisons are presented in Table S5 (Figure 4; Tables S5 and S13).

### 2.4. Leaf Responses and Isolate Aggressiveness Under Controlled Inoculation Conditions

#### 2.4.1. Aggressiveness Following Mycelial-Plug Inoculation of Non-Wounded Leaflets

**Figure 5:**
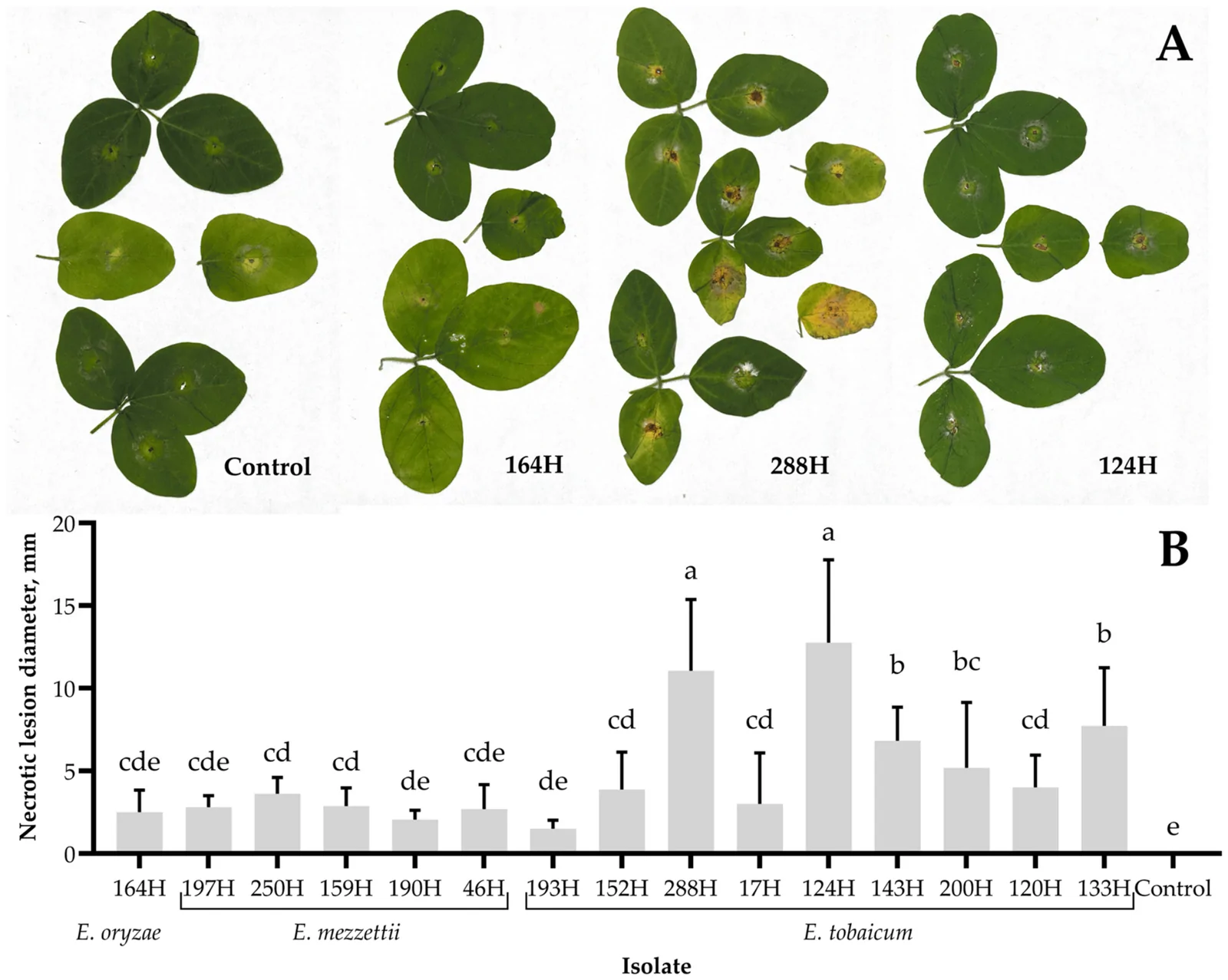
Aggressiveness of *Epicoccum* isolates on soybean leaves following inoculation with mycelial plugs. (**A**) Representative appearance of leaves in the control and after inoculation with *E. oryzae* 164H, *E. tobaicum* 288H, and *E. tobaicum* 124H (from left to right). (**B**) Diameter of the necrotic lesion 7 days after inoculation, calculated as the mean of two mutually perpendicular measurements. Values are presented as the mean ± standard deviation for eight individual plants per treatment (*n* = 8), except for isolates 133H and 143H (*n* = 9); one leaflet was assessed on each plant. Different lowercase letters indicate statistically significant differences among treatments according to Duncan’s multiple range test at *p* < 0.05. A sterile agar plug was placed on control leaflets. No needle puncturing or incision of the leaf blade was performed before placement of the mycelial or sterile agar plugs. Complete numerical data are provided in Table S6.

In the mycelial-plug assay, in which the plug was placed on the intact leaf surface without needle puncturing or incision of the leaf blade, necrotic lesions were observed in all treatments with *Epicoccum* isolates, whereas no necrosis was present in the control treatment. Disease severity differed significantly among treatments (ANOVA, *p* < 0.001; Table S13; Figure 5). The largest lesions were produced by *E. tobaicum* isolates 124H (12.75 ± 5.03 mm) and 288H (11.06 ± 4.32 mm), which did not differ from each other and belonged to statistical group a. High aggressiveness was also observed for *E. tobaicum* 133H (7.72 ± 3.52 mm) and 143H (6.83 ± 2.02 mm), which were grouped in statistical group b.

The smallest necrotic lesion diameter among all studied isolates was recorded for *E. tobaicum* 193H (1.50 ± 0.53 mm). Treatments with *E. oryzae* 164H, *E. mezzettii* 197H, 190H, and 46H, and *E. tobaicum* 193H shared a letter group with the control and did not differ significantly from it. Among *E. mezzettii* isolates, values fell within a relatively narrow range, from 2.06 ± 0.56 mm for 190H to 3.62 ± 0.99 mm for 250H, whereas the single *E. oryzae* isolate examined, 164H, produced lesions 2.50 ± 1.34 mm in diameter. Thus, the greatest inter-isolate variability in this experiment was observed among *E. tobaicum* isolates; species were not compared as separate statistical groups because the numbers of isolates examined were unequal. Complete values and results of multiple comparisons are presented in Table S6 (Figure 5; Tables S6 and S13).

Following inoculation with mycelial plugs, *Epicoccum* cultures were reisolated from symptomatic leaf areas in treatments with all isolates. The cultural and morphological characteristics of the reisolates corresponded to those of the cultures used for inoculation. For all 15 reisolates examined, the rDNA-ITS sequences were completely identical to those of the corresponding original cultures across the compared regions. *Epicoccum* was not isolated from control leaves inoculated with sterile agar plugs.

#### 2.4.2. Aggressiveness of *E. tobaicum* Following Wound-Assisted Conidial Inoculation

**Figure 6:**
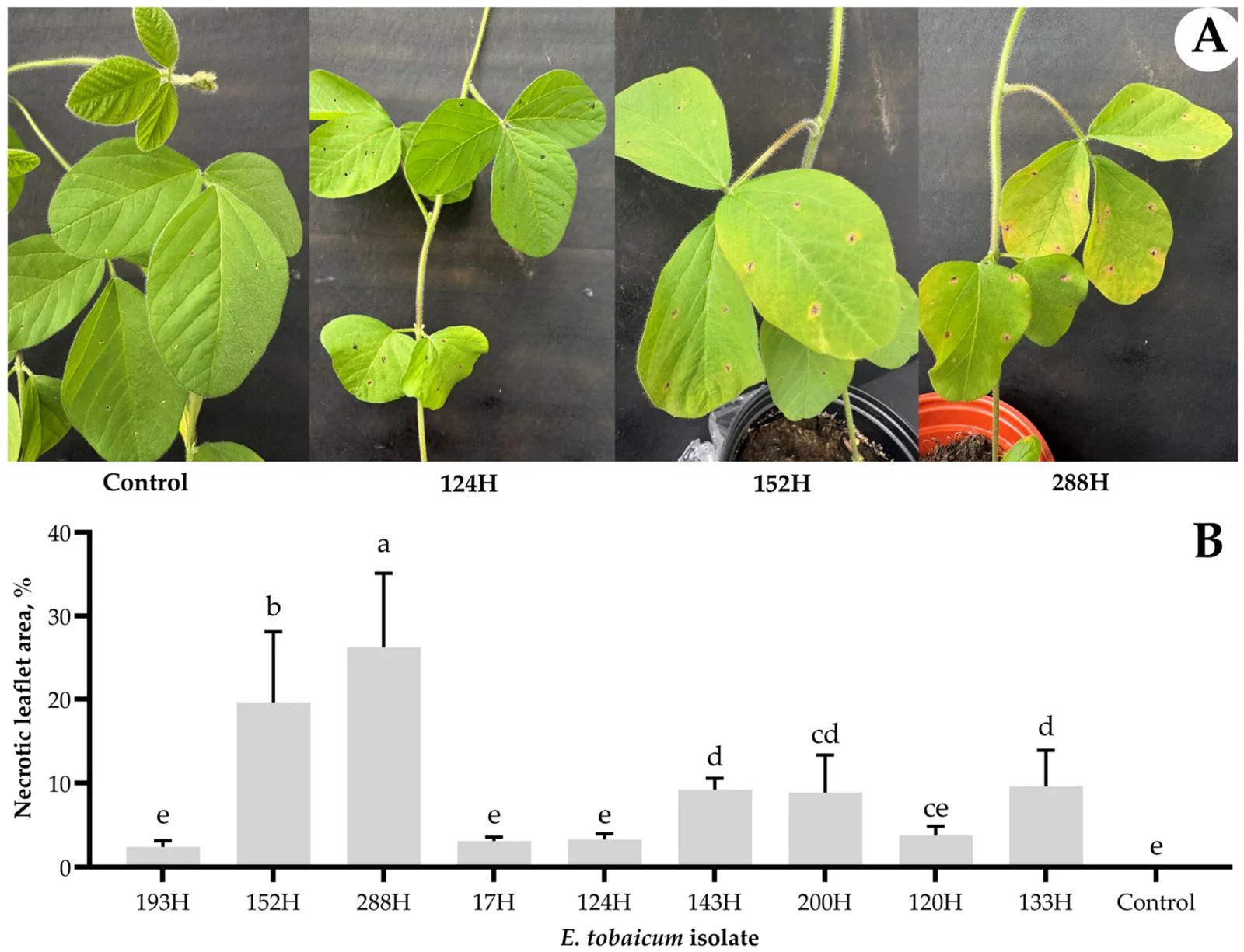
Aggressiveness of *E. tobaicum* isolates on soybean leaves following wound-assisted inoculation with a conidial suspension. (**A**) Representative symptoms in the control and after inoculation with the study isolates. (**B**) Proportion of necrotic area of a leaflet from the first trifoliate leaf at 7 days after inoculation, %. Values are presented as the mean ± standard deviation for six plants; one leaflet was assessed on each plant (*n* = 6). Different lowercase letters indicate statistically significant differences among treatments according to Duncan’s multiple range test at *p* < 0.05. All assessed leaflets, including those of the control treatment, were wounded using the same standardized entomological-needle procedure; inoculated wounds received 20 µL of a suspension containing 1 × 10^6^ conidia/mL, whereas control wounds received 20 µL of sterile water without conidia. The assay therefore compares disease development after facilitated entry and does not evaluate penetration of intact leaf surfaces. Complete numerical data are provided in Table S7.

In the wound-assisted conidial assay, *E. tobaicum* conidial suspensions were applied to standardized needle wounds on soybean leaflets, and disease severity was assessed as the proportion of necrotic leaflet area. Visible lesions were recorded in all treatments with the study isolates, whereas the identically wounded control leaflets treated with sterile water remained symptomless. Disease severity differed significantly among isolates (ANOVA, *p* < 0.001; Table S13; Figure 6), with the proportion of diseased area of the assessed leaflet ranging from 2.42 ± 0.74% for isolate 193H to 26.17 ± 8.93% for 288H. Isolate 288H was the most aggressive and belonged to a separate statistical group, a; the next highest value was recorded for 152H (19.60 ± 8.52%; group b).

For isolates 133H and 143H, diseased areas were 9.63 ± 4.30 and 9.25 ± 1.34%, respectively, and both isolates belonged to group d. The weakest disease development was observed for 193H, 17H, 124H, and 120H; these treatments shared a letter group with the control and did not differ significantly from it. Thus, the wound-assisted conidial assay revealed pronounced intraspecific variability in lesion development within the tested *E. tobaicum* collection. Because the conidia were applied to standardized wounds, these values quantify aggressiveness after facilitated entry and do not measure the ability of the isolates to penetrate an intact leaf surface. *Epicoccum mezzettii* and *E. oryzae* isolates were not included in this experiment because conidia could not be obtained under the cultivation conditions used (Figure 6; Tables S7 and S13).

Following inoculation with conidial suspensions, *Epicoccum* cultures were reisolated from affected leaf areas in all treatments inoculated with the isolates. Their cultural and morphological characteristics corresponded to those of the original *E. tobaicum* cultures. ITS sequences of the nine reisolates examined, one from each inoculated treatment, were completely identical to the sequences of the corresponding original cultures across the compared regions. *Epicoccum* was not isolated from control leaves treated with sterile water.

### 2.5. Effects of Epicoccum Isolates on Soybean Seeds and Seedlings

**Figure 7:**
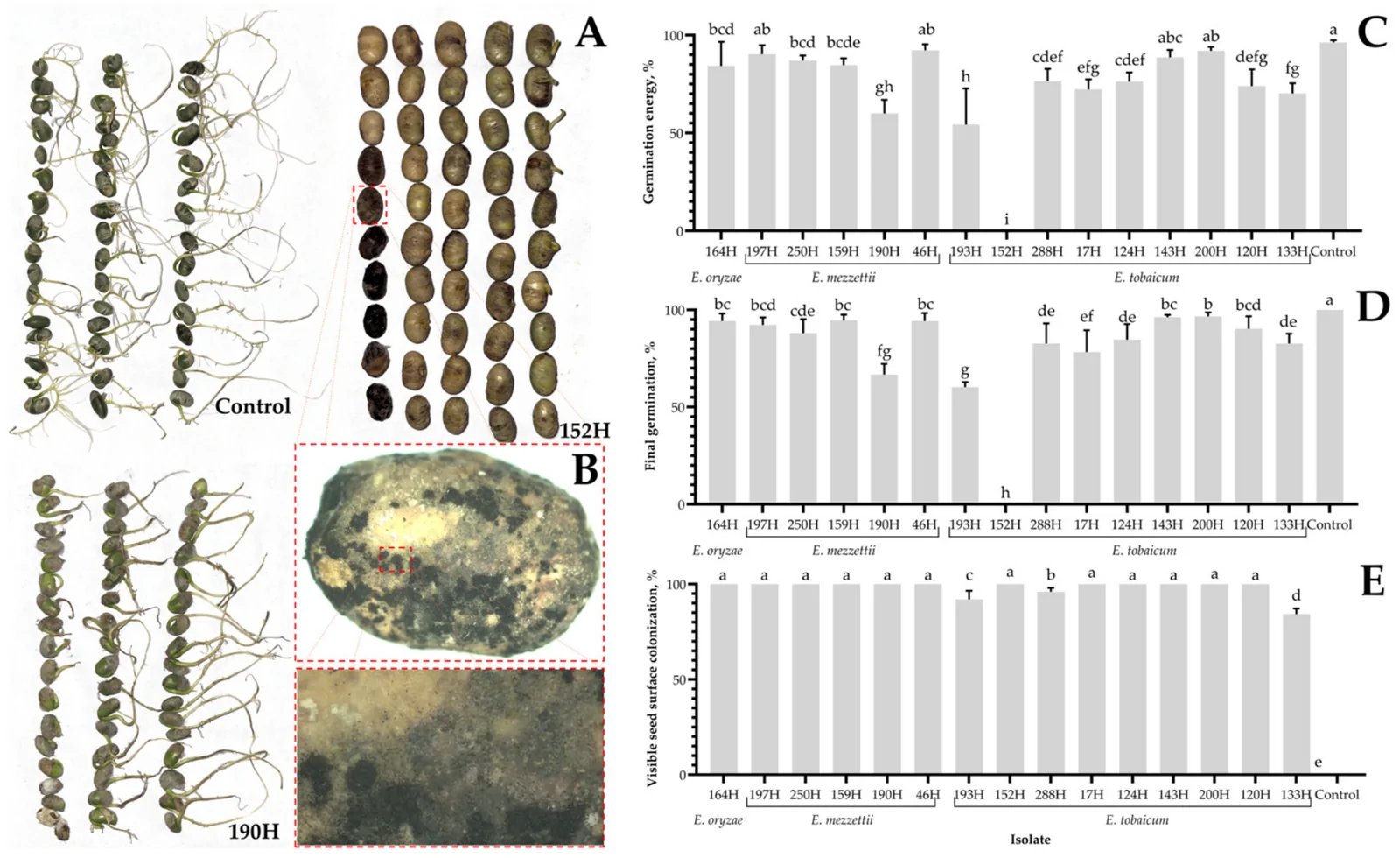
Effects of *Epicoccum* isolates on germination and the condition of soybean seeds and seedlings. (**A**) Representative appearance of seeds and seedlings in the control and after inoculation with *E. mezzettii* 190H and *E. tobaicum* 152H; (**B**) enlarged view of an affected seed in the 152H treatment; (**C**) germination energy; (**D**) germination; (**E**) visible seed surface colonization. Germination energy (**C**) was determined on day 5, whereas final germination (**D**) and visible seed surface colonization (**E**) were determined on day 8 after the start of germination. Values are presented as the mean ± standard deviation of three replicates (*n* = 3); each replicate represented a separately treated lot of 100 seeds. Photographs in (**A**,**B**) were taken on day 8 after the start of germination. Different lowercase letters within each parameter indicate statistically significant differences among treatments according to Duncan’s multiple range test at *p* < 0.05. Complete numerical data are provided in Table S8.

Following artificial inoculation, varying degrees of mycelial colonization were observed on the surfaces of seeds and developing seedlings. *Epicoccum* isolates significantly affected germination energy, final germination, and the proportion of seeds with visually detectable signs of fungal colonization (hereafter, visible seed surface colonization) (ANOVA, *p* < 0.001 for all three variables; Figure 7; Table S13). The strongest adverse treatment effect was observed for *E. tobaicum* 152H: germination energy and final germination were 0.0 ± 0.0%, whereas visible seed surface colonization was 100.0 ± 0.0%. No normal seedlings had developed in this treatment by day 8.

A pronounced reduction in final germination was also caused by *E. tobaicum* 193H (60.3 ± 2.5%) and *E. mezzettii* 190H (66.7 ± 5.5%); germination energy in these treatments was 54.3 ± 18.4% and 60.0 ± 6.9%, respectively. In contrast, final germination remained close to the control level for several isolates, including *E. tobaicum* 200H (96.7 ± 2.1%) and 143H (96.3 ± 1.2%). However, high visible seed surface colonization was not always accompanied by an equivalent reduction in final germination: for example, colonization reached 100% for 200H and 143H despite maintenance of high final germination. In the control, final germination was 100.0 ± 0.0%, and visible seed surface colonization was absent (Figure 7; Tables S8 and S13).

*Epicoccum* cultures were reisolated from seedling tissues in treatments with 14 isolates and from nongerminated seeds in the treatment with isolate 152H. The cultural and morphological characteristics of the resulting cultures corresponded to those of the original cultures. For all 15 reisolates examined, ITS sequences were 100% identical to those of the corresponding original cultures. *Epicoccum* was not isolated from analogous samples of the control treatment.

### 2.6. Extracellular Enzymatic Activity of the Isolates

**Figure 8:**
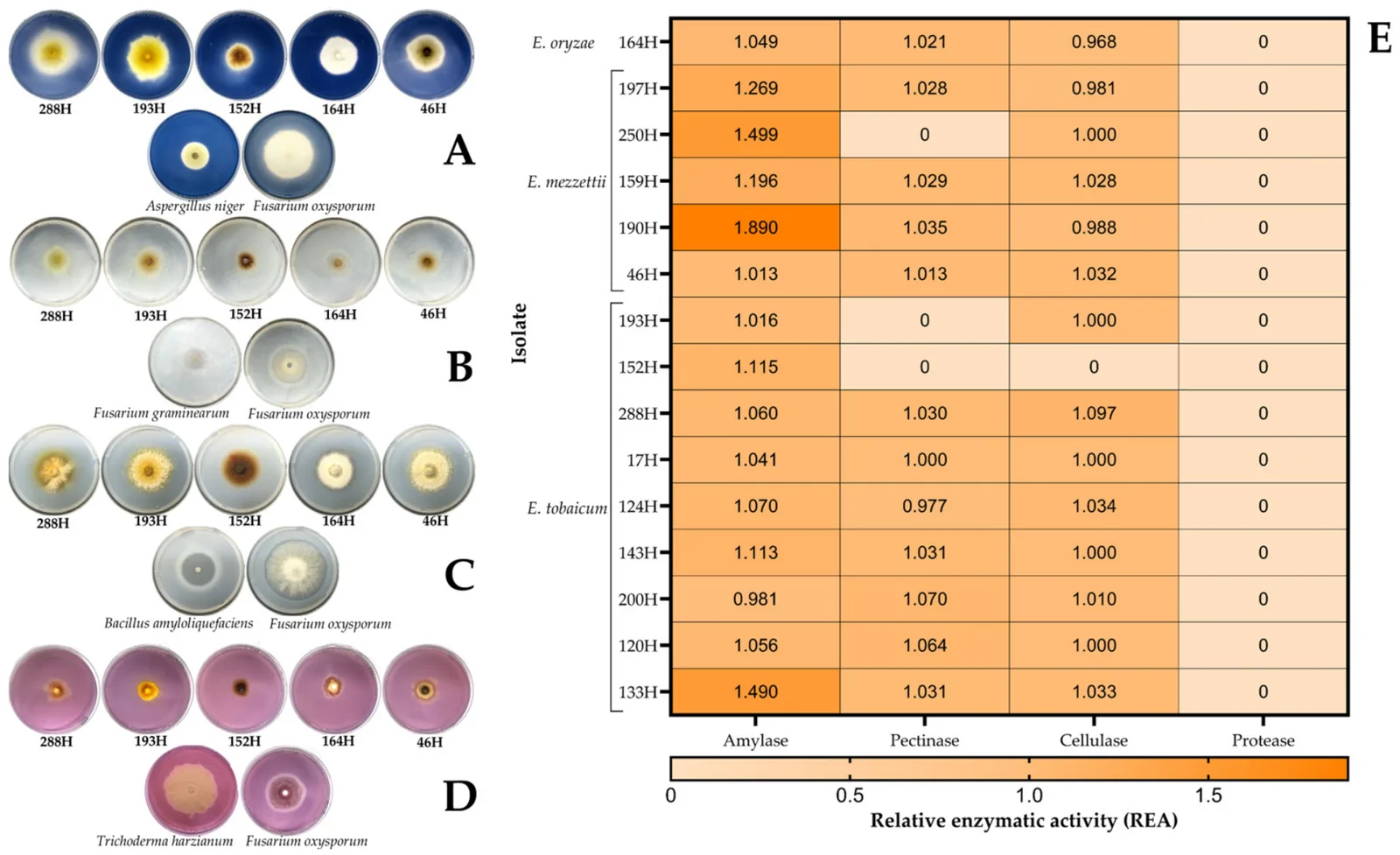
Extracellular enzymatic activity of *Epicoccum* isolates in substrate agar assays. (**A**) Amylolytic activity on starch agar; (**B**) pectinolytic activity on pectin substrate; (**C**) proteolytic activity on milk substrate; (**D**) cellulolytic activity on medium containing carboxymethyl cellulose; (**E**) heatmap of relative enzymatic activity (REA) of the studied isolates. (**C**) includes MBI 600, the positive protease control, and (**D**) includes *Trichoderma harzianum*, the cellulase control; (**A**–**D**) show representative *Epicoccum* isolates and the corresponding positive-control cultures. (**E**) shows mean REA values from three independent replicates; a value of 0 indicates the absence of a detectable hydrolysis zone under the specific in vitro conditions of the assay used and does not indicate the absence of the corresponding enzyme or of its activity during host colonization. Information on the control cultures is provided in Table S9, and complete REA values and results of multiple comparisons are provided in Table S10.

The studied *Epicoccum* isolates showed pronounced variability in amylolytic, pectinolytic, and cellulolytic activities (ANOVA, *p* < 0.001 for all three assays; Figure 8; Table S13). Amylolytic activity was detected in all isolates and ranged from REA = 0.9807 ± 0.0791 for *E. tobaicum* 200H to 1.8896 ± 0.1340 for *E. mezzettii* 190H. The highest value, observed for 190H, corresponded to statistical group a; elevated activity was also recorded for *E. mezzettii* 250H and *E. tobaicum* 133H, which belonged to group b.

Pectinolytic activity was not detected in *E. mezzettii* 250H or *E. tobaicum* 193H and 152H (REA = 0), whereas values for the remaining isolates were approximately 0.98–1.07. The highest numerical value was recorded for *E. tobaicum* 200H (1.0699 ± 0.0915); however, most isolates with detectable activity belonged to the same statistical group, a. Cellulolytic activity was absent only in *E. tobaicum* 152H; values were similar among the remaining isolates, and the highest numerical value was recorded for *E. tobaicum* 288H (1.0967 ± 0.1615). Most isolates with detectable cellulolytic activity also belonged to group a.

Under the conditions of the assay used, proteolytic activity was not detected in the studied *Epicoccum* isolates (REA = 0 in all replicates). At the same time, the positive-control cultures exhibited the expected activity and confirmed proper performance of the corresponding substrate assays. Complete REA values, statistical groups, and information on the positive controls are provided in Tables S9 and S10 (Figure 8; Tables S9, S10 and S13).

### 2.7. Relationships Among Phenotypic Traits of the Isolates

Correlation analysis revealed no statistically significant relationships among leaf aggressiveness, reduction in seed germination, growth rate, and extracellular enzymatic activity after correction for multiple comparisons using the Benjamini-Hochberg procedure (FDR-adjusted *p* > 0.05 for all 15 comparisons). The strongest positive association was observed between necrotic lesion diameter and cellulolytic activity (ρ = 0.5710; *p* = 0.0262); however, it was no longer statistically significant after FDR correction (FDR-adjusted *p* = 0.3686).

The strongest negative association was observed between reduction in seed germination and pectinolytic activity (ρ = −0.4541; *p* = 0.0891; FDR-adjusted *p* = 0.3686). The remaining correlation coefficients likewise did not reach statistical significance after correction for multiple comparisons. Proteolytic activity was not included in the analysis because this trait showed no variability among the studied *Epicoccum* isolates. Complete results of the correlation analysis are provided in Table S11.

### 2.8. In Vitro Fungicide Sensitivity of the Isolates

Sensitivity of the studied *Epicoccum* isolates depended strongly on the active ingredient and showed pronounced variability. The most contrasting response was observed with azoxystrobin: for 14 of the 15 isolates, 50% inhibition of mycelial growth was not achieved even at the highest tested concentration of 1000 µg/mL. These results are therefore right-censored and are reported as EC_50_ > 1000 µg/mL rather than as exact EC_50_ estimates. The exception was *E. tobaicum* 152H, for which the EC_50_ was 0.787 ± 0.212 µg/mL.

**Figure 9:**
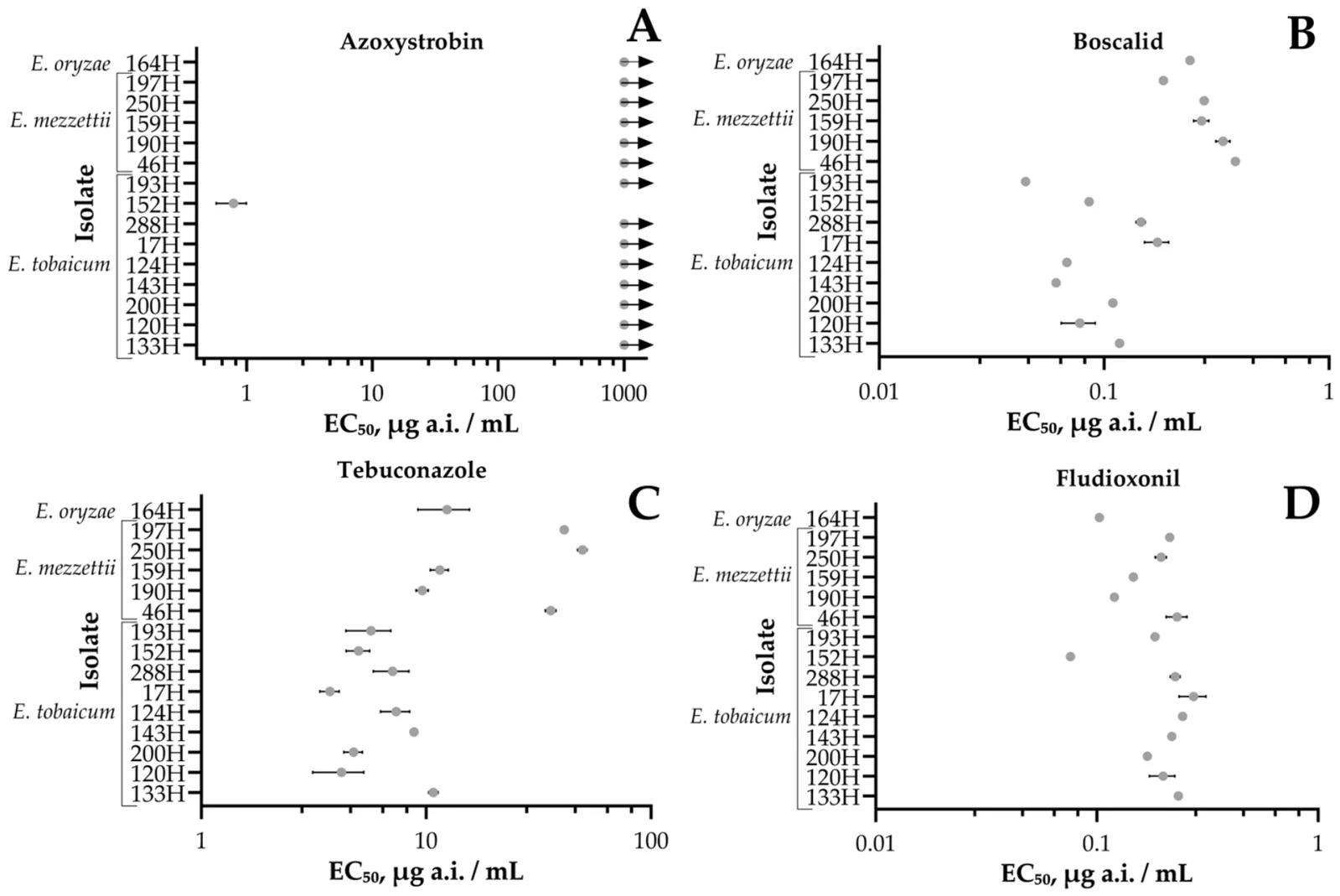
In vitro sensitivity of the studied *Epicoccum* isolates to (**A**) azoxystrobin, (**B**) boscalid, (**C**) tebuconazole, and (**D**) fludioxonil. EC_50_ values are expressed as µg active ingredient per mL of medium. For boscalid, fludioxonil, and tebuconazole, values are presented as the mean ± standard deviation of three independently calculated EC_50_ values (*n* = 3). In all panels, each circle indicates the mean EC50 of an isolate and the horizontal line through the circle indicates ± one standard deviation; where the standard deviation is smaller than the symbol, the line is not visible. For azoxystrobin, a circle with a rightward arrow at 1000 µg/mL indicates a right-censored observation for which 50% inhibition of mycelial growth was not achieved at the highest tested concentration; these are reported as EC_50_ > 1000 µg/mL and are not exact estimates. The isolate order is the same in all panels. Complete EC_50_ values and the results of multiple comparisons are provided in Table S12.

For boscalid, fludioxonil, and tebuconazole, EC_50_ values differed significantly among isolates (ANOVA, *p* < 0.001 for each active ingredient; Figure 9; Table S13). *E. tobaicum* 193H was the most sensitive to boscalid (EC_50_ = 0.0448 ± 0.0016 µg/mL; group k), whereas *E. mezzettii* 46H was the least sensitive (0.3837 ± 0.0026 µg/mL; group a). For fludioxonil, the lowest EC_50_ was recorded for *E. tobaicum* 152H (0.0760 ± 0.0026 µg/mL; group j), and the highest for *E. tobaicum* 17H (0.2736 ± 0.0381 µg/mL; group a).

For tebuconazole, EC_50_ values ranged from 3.735 ± 0.366 µg/mL for *E. tobaicum* 17H to 49.542 ± 2.367 µg/mL for *E. mezzettii* 250H; because these values were below or close to the lowest tested concentration (50 µg/mL), they are approximate estimates obtained by extrapolation (Section 4.8). Overall, pronounced inter-isolate variability in sensitivity was observed for boscalid, fludioxonil, and tebuconazole. Uneven representation of species in the collection precluded interpretation of the detected differences as species-specific. Complete EC_50_ values and results of multiple comparisons are provided in Table S12; representative inhibition of colony growth is shown in Figure S1 (Figure 9 and Figure S1; Tables S12 and S13).

## 3. Discussion

Multilocus analysis based on ITS, *RPB2*, and *TUB2* sequences revealed that the 15 *Epicoccum* isolates obtained from soybean leaves and seeds in Russia represented three phylogenetic lineages corresponding to *E. tobaicum*, *E. mezzettii*, and *E. oryzae*. This finding highlights the importance of multilocus identification for resolving species boundaries within *Epicoccum*, particularly because many isolates previously assigned to the broad *E. nigrum* complex are now recognized as distinct phylogenetic species, while *E. layuense* is considered a synonym of *E. tobaicum* [4]. Accurate species-level identification is particularly important for understanding the phytopathological significance of the genus in soybean, as different *Epicoccum* species have been associated with distinct disease outcomes in this crop. Specifically, *E. sorghinum* and *E. nigrum* have been confirmed as causal agents of foliar diseases, whereas *E. italicum* has been identified as a causal agent of seed disease [10,11,12]. Beyond their association with disease, *Epicoccum* species have also been detected in the endophytic mycobiota of soybean seeds and pods in the United States, and *E. nigrum* has previously been detected in Russian agroecosystems [2,14]. Other members of the genus are known from various plants in Russia [13]. Against this background, the present study combined multilocus identification and phenotypic characterization of 15 *Epicoccum* isolates from soybean leaves and seeds in Russia with comparative genomic analysis of five deliberately selected isolates, providing an integrated assessment of their taxonomic diversity and genomic characteristics.

Whole-genome data were generally consistent with the multilocus placement of the isolates: the highest FungANI similarity was observed among the three *E. tobaicum* genomes, the lower similarity between *E. mezzettii* and *E. tobaicum*, and the lowest similarity in comparisons of *E. oryzae* with the other genomes. Because the applicability of fixed ANI thresholds for delineating fungal species depends on the particular phylogenetic group, these values should be regarded as an additional measure of genomic relatedness rather than as an independent taxonomic criterion [15,16]. The comparative genomic analysis is exploratory and hypothesis-generating: its contribution is to delimit the three lineages at the whole-genome level and to provide a first inventory of their predicted functional repertoires, rather than to define species-diagnostic features. The five genomes examined had similarly sized predicted secretomes and repertoires of candidate effectors, CAZymes, PHI-base homologs, and potential BGCs. Thus, differences in the overall sizes of these predicted functional repertoires did not provide a simple explanation for the phenotypic variation observed among the isolates. All of these categories are computational predictions: PHI-base matches indicate sequence homology to experimentally characterized pathogen-host interaction proteins in other systems and do not constitute functional validation in *Epicoccum*, and the candidate effectors and CAZymes were not experimentally tested. The most notable validated isolate-associated copy-number difference was the expansion of the spherulin/germin-like Cupin_1 orthogroup OG0000216 in *E. tobaicum* isolate 152H. Although the involvement of a Cupin_1-containing protein in fungal virulence has been demonstrated in another phytopathogenic fungus [17], the biological roles of the OG0000216 proteins in *Epicoccum* remain unknown. Their expression and function were not experimentally examined, and no causal relationship between the OG0000216 expansion and the leaf or seed phenotypes of isolate 152H has been established. Accordingly, this expansion should be considered a validated genomic feature of the examined isolate and a hypothesis for future functional studies, not a demonstrated mechanism of pathogenicity. Furthermore, because only one genome each of *E. mezzettii* and *E. oryzae* and three purposively selected *E. tobaicum* genomes were compared, these isolate-level genomic differences should not be generalized to entire species.

The studied isolates exhibited pronounced cultural and physiological heterogeneity. Growth rates differed among isolates within each culture medium, and their ranking based on mean values also changed among media. High intraspecific phenotypic variability has previously been demonstrated for the *E. nigrum* complex and other molecularly identified members of the genus [6,18]. All nine *E. tobaicum* isolates sporulated on PDA, whereas conidia could not be obtained from the *E. mezzettii* and *E. oryzae* isolates examined under the cultivation conditions used. Taken together, these results confirm that cultural characteristics are useful for characterizing individual isolates, but their pronounced variability limits the use of morphology as a stand-alone tool for species-level identification of *Epicoccum* [4].

The strongest evidence of leaf-damaging capacity was obtained for individual *E. tobaicum* isolates. In the mycelial-plug assay, performed without needle puncturing of the leaf blade, isolates 124H and 288H produced the largest necrotic lesions, whereas wound-assisted conidial inoculation resulted in the most pronounced disease symptoms with isolates 288H and 152H. The ranking of *E. tobaicum* isolates differed between the two experiments, which differed in inoculum form and mode of application, in whether the leaf tissue was wounded, and in the disease variable measured. The individual contributions of these factors were not evaluated. The pronounced inter-isolate variability is consistent with the results of studies of *Epicoccum* on maize and Italian ryegrass [6,7]. The pathogenicity of *E. tobaicum* has previously been confirmed on flowering cherry, oat, and Italian ryegrass [7,19,20], and the species was recently established as the causal agent of a foliar disease of urdbean (*Vigna mungo*), which, like soybean, belongs to the *Fabaceae* [21]. In the present study, however, the conidial suspensions were applied to standardized needle wounds. Because wounding bypasses the cuticle and other natural penetration barriers, this experiment should be interpreted as a comparison of aggressiveness after facilitated entry rather than as evidence that the isolates can initiate infection on intact leaves. Reisolation of *Epicoccum* from symptomatic tissues and the identity of the ITS sequences with those of the corresponding inoculated cultures support an association between the inoculated isolates and the resulting necrosis under these controlled conditions, but they do not establish natural penetration efficiency. We therefore interpret these experiments as evidence of pathogenic potential under controlled conditions, and confirmation of disease development following inoculation of intact leaflets is required before natural leaf infection by these isolates can be inferred. In addition, several *E. tobaicum* isolates did not differ statistically from the control, indicating substantial intraspecific variability and precluding extrapolation of high aggressiveness to the species as a whole.

The data obtained for *E. mezzettii* and *E. oryzae* permit interpretation at the isolate level only. Following inoculation with mycelial plugs, individual isolates produced small necrotic lesions; however, several *E. mezzettii* treatments and the single *E. oryzae* isolate examined did not differ statistically from the control, and conidial inoculation could not be performed for these species. Therefore, the available results are insufficient to characterize *E. mezzettii* and *E. oryzae* as established causal agents of soybean leaf spot. This is particularly noteworthy for *E. mezzettii*, which has previously been regarded primarily as a plant-associated or antagonistic fungus; representatives of this species have been shown to suppress phytopathogenic fungi in vitro [5,22]. Nevertheless, the pathogenicity of *E. oryzae* has been confirmed on Italian ryegrass [7]. These differences emphasize the ecological plasticity of the genus and the need to evaluate the specific interaction between an isolate and a particular host plant.

Artificial seed inoculation revealed pronounced inter-isolate variability. Most isolates actively colonized the seed surface; however, the intensity of visual colonization did not always correspond to a reduction in germination. Thus, after inoculation with *E. tobaicum* 143H and 200H, visible seed surface colonization reached 100%, whereas final germination remained above 96%. In contrast, no normal seedlings were observed following inoculation with *E. tobaicum* isolate 152H, and final germination was reduced following inoculation with *E. tobaicum* isolate 193H and *E. mezzettii* isolate 190H. The seed inocula were standardized by the counted number of mycelial fragments, whereas fragment viability and fungal biomass were not independently quantified. Consequently, the between-isolate differences in final germination and visible seed surface colonization may reflect both intrinsic biological differences among the isolates and differences in the viable inoculum or biomass represented by an equal fragment count. The seed experiment is therefore interpreted as a comparison of treatment effects under a standardized protocol rather than as a direct quantitative ranking of intrinsic seed aggressiveness. Importantly, five study isolates representing all three species identified were originally obtained from naturally collected soybean seeds, confirming their natural association with seed material. These observations are consistent with previous reports of *Epicoccum* in the mycobiota of soybean seeds and pods [8,9,14]. However, the ability to colonize seeds naturally does not necessarily imply a pronounced effect on seed viability, as demonstrated by differences among the studied isolates. The ability of *E. italicum* to cause soybean seed disease and reduce germination has been demonstrated experimentally [12]. Our results extend these findings by showing that individual *E. tobaicum* and *E. mezzettii* isolates can also substantially reduce germination following artificial inoculation. However, separate experiments using naturally infected seed material are required to demonstrate natural transmission of infection from seed to seedling.

Enzymatic assays revealed differences among isolates. Amylolytic activity was recorded in all isolates, whereas pectinolytic and cellulolytic activities were absent in some isolates, and proteolytic activity was not detected under the conditions used. Inter-isolate variability in hydrolytic enzyme activity has also previously been demonstrated for *Epicoccum* species [6,23]. *Epicoccum tobaicum* 152H is particularly illustrative: no detectable pectinolytic or cellulolytic activity was observed in the specific substrate-agar assays used here, yet this isolate produced pronounced leaflet damage in the wound-assisted conidial assay, and no normal seedlings were observed after artificial seed inoculation with it. These plate assays provide semiquantitative measurements under defined in vitro conditions of medium composition, pH, temperature, and incubation time; the absence of a detectable hydrolysis zone does not demonstrate the absence of the corresponding genes or enzymes, nor the absence of enzyme activity during host colonization. Accordingly, the present data do not support a direct mechanistic link between any single in vitro enzyme phenotype and aggressiveness, and the correspondence, or lack of correspondence, between the two should not be interpreted mechanistically. Correlation analysis likewise revealed no statistically significant relationships among leaf aggressiveness, reduction in seed germination, growth rate, and enzymatic activity after FDR correction. The absence of statistically significant correlations in the collection of 15 isolates does not exclude biological relationships among the traits. The predictive utility of individual cultural and enzymatic variables was not tested in the present study.

Pronounced inter-isolate variability was also observed in fungicide sensitivity. Boscalid and fludioxonil exhibited relatively low EC_50_ values, whereas sensitivity to tebuconazole varied over a much wider range. Reduced sensitivity to tebuconazole has previously been reported among *E. layuense* isolates from maize [6]. Substantial differences in the response of members of the genus to demethylation inhibitor fungicides have also been reported in other pathosystems [24]. In contrast, *E. nigrum* causing soybean leaf blight in China has been shown to be considerably more sensitive to tebuconazole [11]. Therefore, sensitivity metrics for one species or local population should not be extrapolated to the genus *Epicoccum* as a whole.

The low apparent in vitro sensitivity of most isolates to azoxystrobin warrants particular attention: for 14 of the 15 isolates, 50% inhibition of mycelial growth was not achieved even at the highest tested concentration of 1000 µg/mL, so these values are right-censored rather than exact estimates, whereas *E. tobaicum* 152H was markedly more sensitive. This phenotype should not be interpreted as proven resistance to QoI fungicides. Sensitivity assessment based on mycelial growth may be affected by alternative respiration, and the effects of SHAM on assay results depend on the pathogen and experimental system [25]. In studies of QoI resistance, molecular confirmation of the mechanism usually requires additional analysis of the target, for example, mutations in the *cytB* gene [26]. Because SHAM was not used and molecular resistance mechanisms were not investigated in the present study, the values obtained characterize the apparent phenotypic sensitivity of mycelial growth under the conditions of this in vitro assay; they do not establish QoI resistance in field populations and do not support recommendations for field-level disease management. Similarly, the low EC_50_ values for boscalid and fludioxonil indicate activity against the tested isolates; however, the efficacy of products containing these compounds requires further evaluation under field conditions.

Taken together, the results show that the biological properties of soybean-associated *Epicoccum* are largely determined by the individual isolate and the nature of its interaction with plant tissues. This finding is consistent with the view of *Epicoccum* as an ecologically plastic genus whose members may exhibit endophytic, saprotrophic, antagonistic, or phytopathogenic lifestyles [5]. The present study has several limitations that define the scope of inference. The initial sampling was symptom-directed and was not designed to determine the prevalence or population structure of *Epicoccum* in Russia; the species were unevenly represented, with only a single isolate of *E. oryzae* included; conidial leaf inoculation was performed only for *E. tobaicum* and involved prior wounding of the leaf tissue; the seed assay was based on artificial inoculation, and the viability and biomass of the inoculum were not independently quantified; the whole-genome comparison included five deliberately selected isolates, with one genome each for *E. mezzettii* and *E. oryzae*; the predicted effectors, CAZymes, PHI-base homologs, BGCs, and the OG0000216 expansion were not functionally validated; and fungicide sensitivity was assessed only in vitro. These limitations do not negate the observed isolate-level phenotypic differences, but they preclude inference about natural infection efficiency, species-wide genomic characteristics, natural seed transmission, or field resistance. Nevertheless, the combination of multilocus identification, comparative genomics, and several independent phenotypic assays broadens our understanding of the diversity and potential phytopathological significance of *Epicoccum* associated with soybean in Russia.

Future research should focus primarily on confirming pathogenicity on intact plants, assessing natural colonization and seed transmission, and expanding monitoring of populations and their fungicide sensitivity. Functional validation of the genomic differences identified will further clarify the mechanisms underlying the aggressiveness of individual isolates.

## 4. Materials and Methods

### 4.1. Origin of Material, Isolation, and Culture Maintenance

Symptomatic leaves were collected during phytosanitary monitoring of the fungal disease complex of soybean (*Glycine max* (L.) Merr.), and seeds from the Khabarovsk region, the Amur region, the Jewish Autonomous region, and the Tula region were analyzed in 2024–2026. The monitoring was not designed specifically to search for *Epicoccum*. Leaves with necrotic spots and seeds with seed-coat discoloration were selected for mycological analysis. Samples were labeled with the collection location, year, and type of plant material. After removal from the plants, leaves were transported to the laboratory in separate paper bags containing sterile filter paper and stored at 4 °C until analysis. The maximum storage period for leaf samples before culture isolation was 5 days. At least 100 g of seeds from each sample were likewise stored under dry conditions at room temperature. The final study collection comprised 15 *Epicoccum* isolates: 10 from the Khabarovsk region, three from the Jewish Autonomous region, one from the Amur region, and one from the Tula region; 10 isolates were recovered from leaves and five from seeds. Nine isolates were obtained in 2024, five in 2025, and one in 2026. The sampling scheme was symptom-directed and was not designed to estimate the prevalence of *Epicoccum*.

To isolate fungi from symptomatic leaves, 1 × 1 cm pieces were excised with a sterile scalpel from the margin between affected and healthy tissue, treated with 70% ethanol for 5 s and 1% sodium hypochlorite solution for 2 min, rinsed three times with sterile distilled water (SDW) to remove residual disinfectants, and dried on sterile filter paper. Seeds were surface-sterilized in the same manner, except that after sterilization they were cut in half with a sterile scalpel under aseptic conditions.

The prepared material was placed in Petri dishes containing water agar (WA) supplemented with streptomycin sulfate and chloramphenicol at 100 mg/L each (Central Drug House, New Delhi, India) and incubated at 20 ± 1 °C in a KB 23 incubator (BINDER GmbH, Tuttlingen, Germany). After 5–6 days, mycelial growth was observed; a hyphal fragment was excised with a sterile needle from the colony margin at a site not in contact with the original leaf or seed piece and transferred to potato dextrose agar (PDA; Central Drug House, New Delhi, India) supplemented with streptomycin sulfate and chloramphenicol at 100 mg/L each (Central Drug House, New Delhi, India), followed by incubation under the conditions described above. This subculturing step was repeated three times to obtain pure cultures. Purified isolates were stored on agar at +4 °C and in 20% glycerol at −80 °C and were transferred to fresh PDA before experiments.

The collection used in this study comprised 15 *Epicoccum* isolates obtained during mycological analysis of the collected soybean leaves and seeds (Table 1). Preliminary assignment of the cultures to the genus was based on a combination of cultural and morphological characteristics. Colony growth and pigmentation, development of aerial mycelium, and, when sporulation occurred, formation of sporodochia and darkly pigmented multicellular conidia with transverse and longitudinal septa and a rough surface, characteristic of the epicoccoid morph of *Epicoccum* species, were recorded [3,4]. Final genus- and species-level identification of the isolates was established by multilocus analysis of ITS, *RPB2*, and *TUB2* sequences.

### 4.2. Molecular Identification and Multilocus Phylogenetic Analysis

Final identification of the isolates was performed using ITS, *RPB2*, and *TUB2* sequences. DNA was extracted from 7-day-old mycelium grown on PDA using the Phytosorb DNA extraction kit (Sintol LLC, Moscow, Russia) according to the manufacturer’s protocol. Amplification reaction mixtures contained 5 µL of 5× Master Mix (5× MasDDTaqMIX-2025, Dialat, Moscow, Russia), 2.5 µL of each primer from a 10 µM working solution, corresponding to a final concentration of 1.0 µM for each primer, 5 ng of template DNA, and PCR-grade water (Sintol LLC, Moscow, Russia) to a total volume of 25 µL. The rDNA-ITS region was amplified in a T100 thermal cycler (Bio-Rad, Hercules, CA, USA) using primers ITS5/ITS4, the *RPB2* fragment using RPB2-5F2/fRPB2-7cR, and *TUB2* using Btub2Fd/Btub4Rd, in accordance with previously published protocols for *Didymellaceae* and *Epicoccum* [3,22,27].

Amplicons were separated by electrophoresis in a 1.5% agarose gel, stained with ethidium bromide in 0.5× TBE buffer, and visualized using a Gel Doc XR+ system (Bio-Rad, Hercules, CA, USA). PCR fragments were excised and purified using the ColGen kit (Sintol LLC, Moscow, Russia) according to the manufacturer’s instructions. Purified PCR products were sequenced by the Sanger method using the BigDye Terminator v3.1 Cycle Sequencing Kit (Life Technologies Thermo Fisher, Waltham, MA, USA) and a 3730 DNA Analyzer (Thermo Fisher Scientific, Waltham, MA, USA) at Evrogen LLC (Moscow, Russia).

Reference sequences of *E. tobaicum, E. mezzettii, E. oryzae, E. italicum*, and *E. nigrum* were retrieved from GenBank, with priority given to type, ex-type, and other authenticated strains; *E. sorghinum* CBS 179.80 was used as the outgroup. Species assignment was based on the position of isolates in the multilocus phylogeny rather than on sequence similarity at any single locus. Accession numbers for all study and reference sequences are provided in Supplementary Table S1. Each locus was aligned separately in MAFFT v7.490 and trimmed at the terminal regions [28]. Final alignment lengths were 453 bp for ITS, 596 bp for *RPB2*, and 264 bp for *TUB2*; the concatenated dataset comprised 1313 positions. The ITS and *TUB2* datasets each contained 32 sequences, whereas *RPB2* contained 30 sequences; missing *RPB2* data for two reference strains were treated as missing data.

A maximum-likelihood tree was inferred in IQ-TREE v3.1.3 [29]. The partitioning scheme and substitution models were selected with ModelFinder in MFP + MERGE mode; ITS and *TUB2* were merged into a partition with the TN + I model, whereas K2P + G4 was used for *RPB2* [30]. Node support was assessed with 1000 SH-aLRT replicates and 1000 UFBoot replicates [31,32]. The tree was visualized in iTOL [33]. Taxonomic interpretation followed the revision in which *E. layuense* is treated as a synonym of *E. tobaicum* [4].

### 4.3. Whole-Genome Sequencing, Assembly, Quality Control, and Genome Annotation

Five isolates were purposively selected for comparative whole-genome analysis to represent all three lineages identified in the multilocus phylogeny and to encompass phenotypic variation observed among the study isolates: *E. mezzettii* 46H, *E. oryzae* 164H, and *E. tobaicum* 152H, 193H, and 288H. Three *E. tobaicum* isolates were selected to capture phenotypic variation within the most frequently represented lineage in the collection. The selection was non-random and was intended to compare the selected isolates, not to estimate population-level or species-wide genomic diversity. Because *E. mezzettii* and *E. oryzae* were each represented by only one sequenced isolate, genomic features observed in these isolates cannot be regarded as representative of their respective species.

Paired-end whole-genome sequencing was performed on the DNBSEQ-G400 platform. *De novo* assembly was performed in SPAdes v4.3.0, and contigs at least 500 bp long were retained for subsequent analysis [34]. Assemblies were screened for potential biological and technical contamination using Kraken2 v2.17.1, Tiara v1.0.3, NCBI FCS-adaptor v0.5.5, and FCS-GX v0.5.5+galaxy2 [35,36,37]. Confirmed foreign, adapter, and vector sequences were removed or trimmed. Cleaned assemblies and their corresponding final predicted protein sets were used for the final comparative analyses; annotations of regions affected by the final cleaning step were synchronized with the final sequences. Support of the final assemblies by the raw reads was assessed after mapping the reads to the corresponding assembly using bwa-mem2 v2.3 and samtools v1.24.

For each genome, a de novo repeat library was independently constructed using RepeatModeler v2.0.9, followed by soft masking with RepeatMasker v4.2.4 [38,39]. Structural annotation was performed in Funannotate v1.8.15 using the predict workflow, with the fungal organism setting (organism fungus), the pezizomycotina BUSCO training dataset (busco_db pezizomycotina), UniProt/Swiss-Prot protein evidence, and a minimum predicted protein length of 50 amino acids [40]. The pezizomycotina dataset was used only during predictor training. Final assembly and proteome completeness were assessed with BUSCO v6.1.0 using the dothideomycetes_odb10 dataset (*n* = 3786) [41]. Functional annotation of predicted proteins was performed using InterProScan v5.78-109.0 and eggNOG-mapper v2.1.15 with DIAMOND v2.0.15; the results were integrated into the final Funannotate annotations [42,43,44].

For conservative identification of the classical secretome, complete proteomes were first analyzed with DeepTMHMM v1.0 Academic [45]. Only proteins classified as SP by DeepTMHMM were additionally analyzed with SignalP 6.0; proteins confirmed by SignalP as Sec/SPI were included in the consensus classical secretome [46]. EffectorP v3.0 was run only on proteins in this consensus secretome [47]. CAZymes were annotated using run_dbCAN/dbCAN v5.2.9; proteins supported by at least two dbCAN components were considered high-confidence, and secreted high-confidence CAZymes were defined as the intersection of this set with the consensus secretome [48].

PHI-base homologs were searched using DIAMOND v2.2.5 in sensitive mode [49]. Matches with at least 30% identity, at least 50% query coverage, and an E-value ≤ 1 × 10^−5^ were retained; for each protein, only the single best match based on the highest bit score was retained. PHI-base matches were interpreted as evidence of homology rather than experimental confirmation of the function of the corresponding *Epicoccum* protein. Potential biosynthetic gene clusters (BGCs) were predicted using antiSMASH v8.0.4 in fungal mode with gene prediction disabled; the number of unique predicted genomic regions was used for comparisons [50].

Whole-genome nucleotide similarity was assessed pairwise in FungANI v0.1.0 using a 10% genome sample, 1000-bp windows with 500-bp overlap, and a minimum identity threshold of 80% [16]. The symmetric value for each pair was calculated as the mean of the forward and reverse comparisons after filtering windows by the identity threshold. Orthogroups were inferred in OrthoFinder v3.1.5 using DIAMOND v2.2.5, MAFFT v7.526, and FastTree v2.2.0 [51,52]. Candidate differences in presence and copy number were checked on the basis of protein length, position relative to contig boundaries, and direct tBLASTn searches (NCBI BLAST+ v2.16.0) [53]; gene trees, duplication events, raw-read support, and similarity of flanking regions were additionally analyzed for key candidates. Absence of a protein from the predicted proteome was not interpreted as a genomic deletion without additional verification.

Raw whole-genome sequencing reads have been deposited in the NCBI Sequence Read Archive under BioProject PRJNA1521810. BioSample, SRA Run, and locus-tag prefixes for individual isolates are provided in Supplementary Table S2. The final genome assemblies have been submitted to NCBI GenBank under the same BioProject; their accession numbers had not been assigned at the time of submission of this revised manuscript and will be provided to the editorial office before publication.

### 4.4. Cultural-Morphological Characterization and Colony Growth Rate

#### 4.4.1. Colony and Conidial Morphology

Cultural characteristics of the studied *Epicoccum* isolates were examined on potato dextrose agar (PDA), malt extract agar (MEA), and oatmeal agar (OA). The use of this set of media followed approaches employed for cultural-morphological characterization of members of the family *Didymellaceae* [3,6]. All media were sterilized by autoclaving at 121 °C for 30 min. For PDA, a commercial potato dextrose agar medium (Central Drug House, New Delhi, India) was prepared using 39 g of premixed powder and water to a final volume of 1000 mL according to the manufacturer’s instructions. Malt extract agar (MEA) was prepared per 1 L of medium using 20.0 g malt extract, 15.0 g agar, and water to a final volume of 1000 mL [54]. Oatmeal agar (OA) was prepared from a filtered oatmeal decoction. To prepare 1 L of medium, 30.0 g of rolled oats were boiled in 1000 mL of distilled water for 30 min. The resulting mixture was filtered through gauze to remove solid particles. Agar (20.0 g) was added to the filtrate, and the volume was restored to 1000 mL with distilled water [55]. After autoclaving and cooling to 50 °C, 20 mL of medium was dispensed into 90-mm Petri dishes using a Levo Plus electronic dispenser (DLAB Scientific Co., Ltd., Beijing, China) in a sterile laminar-flow cabinet.

After the media had solidified, a 5-mm agar plug with mycelium from the actively growing margin of a 5-day-old PDA culture was transferred to the center of each plate. Cultures were incubated at 25 °C in a KB 23 incubator. Three plates were used for each isolate-medium combination. Surface color, aerial mycelial structure, colony topography, margin characteristics, zonation, visible medium pigmentation, and sporulation were recorded on days 10 and 20. Colonies were photographed from above. When colony figures were prepared, images from the two time points were combined within a single panel: the left half corresponded to day 10 and the right half to day 20; the image-combination procedure is specified in the figure captions.

The morphology of conidia formed on PDA was examined in water-mounted preparations. To prepare the slides, material containing mycelium and conidia was transferred to water and suspended. The resulting suspension was filtered through gauze to reduce the content of large fragments and mycelial aggregates. A drop of the filtrate was placed on a microscope slide, covered with a coverslip, and gently pressed to distribute the material in a thin layer, producing a squash preparation. Water was used as the mounting medium.

Preparations were examined by light microscopy using a PrimoStar microscope (Carl Zeiss, Oberkochen, Germany) at 200× magnification. Conidial shape and pigmentation, the presence and arrangement of septa, and surface characteristics were described. Digital micrographs were acquired with an Axiocam 208 color camera (Carl Zeiss, Germany) mounted on the microscope using ZEN 3.4 software (blue edition; Carl Zeiss, Germany). Conidia of nine *E. tobaicum* isolates formed on PDA after 14 days of cultivation were used for morphometric analysis. For each isolate, material was collected from two Petri dishes, and a total of 30 conidia were measured. Conidial length and width were measured using ImageJ 1.54g (National Institutes of Health, Bethesda, MD, USA). For each isolate, the range and mean ± standard deviation were calculated separately from measurements of 30 conidia; dimensions were expressed in micrometers. Scale bars were included in the micrographs. Failure to obtain conidia was reported only with respect to the media and cultivation conditions used.

#### 4.4.2. Determination of Colony Growth Rate

On day 7, the diameter of each colony was measured in two mutually perpendicular directions using an ADA Mechanic 150 PRO digital caliper (ADA Instruments, Shenzhen, China), and the values were averaged. The mean rate of increase in colony diameter was calculated using the following formula [56]:

*V* = ((*D*_7_ − *D*_0_))/7, (1)

where *V* is the mean rate of increase in colony diameter, mm/day; *D*_7_ is the mean diameter on day 7, mm; and *D*_0_ = 5 mm. The individual plate was the observational unit; three values were obtained for each isolate × medium combination. The two directional measurements of a single colony were averaged to obtain one recorded value. The results are presented in Table S5.

### 4.5. Assessment of Soybean Leaf Responses Under Controlled Artificial Inoculation

Seeds of soybean cultivar Pripyat were sown in a peat-perlite substrate (Veltorf, Vologda, Russia) in 0.5-L plastic containers (AgrofloRaPak, Vologda, Russia). Plants were maintained in a greenhouse at 28/22 °C (day/night) under natural light with a photoperiod of approximately 14 h and were watered as needed. The temperature regime corresponded to soybean growing conditions described previously [56]. Plants with 3-4 fully expanded trifoliate leaves were used for inoculation; inoculated leaflets were visually healthy and comparable in developmental stage within the corresponding leaf position. Experiments with mycelial plugs and conidial suspensions were conducted separately. A separate negative control was included in each experiment.

#### 4.5.1. Inoculation with Mycelial Plugs

The experiment included all 15 *Epicoccum* isolates and a negative control. Plugs 5 mm in diameter were cut with a sterile cork borer from the peripheral region of colonies grown on PDA for 5 days. One plug was placed mycelium-side down on the central region of the leaf blade and pressed with a sterile spatula to ensure contact between the inoculum and the tissue. No needle puncturing or incision of the leaf blade was performed before placement of the mycelial or sterile agar plugs. A sterile agar plug was placed on the leaflets of control plants instead of a mycelial plug. Control plants were maintained under the same conditions as plants inoculated with mycelial plugs. After inoculation, the plants were covered with polyethylene bags to provide high humidity, and the bags were removed after 3 days of incubation. All other growing conditions remained unchanged until the end of the experiment. Necrotic lesion development on the assessed leaflets was evaluated 7 days after inoculation. Necrotic lesion diameter was used as the quantitative measure of disease. No symptoms were observed throughout the experiment on control leaflets receiving sterile agar plugs.

Before measurement, the agar plug was removed with forceps, after which the boundaries of the necrotic lesion on the leaf tissue were determined. Necrotic lesion diameter was defined by the outer boundaries of visibly necrotic tissue; the surrounding chlorotic zone was excluded from the measurement. Measurements were made with an ADA Mechanic 150 PRO caliper in two mutually perpendicular directions and averaged [26]. The mean of the two measurements was used as a single observation for the corresponding plant. The two measurements of one lesion were not treated as separate replicates. In the absence of visible necrosis, a value of zero was recorded. The parameter was expressed in millimeters. The diameter of the agar plug was not subtracted from the measured necrotic lesion diameter. Lesion size was determined independently of the area in contact with the inoculum; therefore, values could be less than 5 mm.

Eight individual soybean plants were used for each isolate and for the control treatment, except for isolates 133H and 143H, for which nine plants were assessed. One leaflet was assessed on each plant. In the experimental treatments, one mycelial plug was placed on the assessed leaflet, whereas in the control treatment a sterile agar plug of the same size was used. An individual plant was considered the experimental unit; each treatment comprised eight biological replicates (*n* = 8), except for isolates 133H and 143H (*n* = 9). Plants were randomly assigned to treatments and randomly positioned in the greenhouse.

#### 4.5.2. Wound-Assisted Inoculation with a Conidial Suspension

Nine *E. tobaicum* isolates, 193H, 152H, 288H, 17H, 124H, 143H, 200H, 120H, and 133H, were used to compare isolate aggressiveness following wound-assisted conidial inoculation. *Epicoccum mezzettii* and *E. oryzae* isolates were not included in this experiment because conidia of these species could not be obtained under the cultivation conditions used. Conidia were collected from 15-day-old PDA cultures into sterile water, suspended, and filtered through gauze to remove mycelial fragments. Plant growth conditions and developmental stage at the time of inoculation were as described in Section 4.5. Conidial concentration was determined by direct counting in a hemocytometer. After counting, the initial suspension was diluted with sterile water to a final concentration of 1 × 10^6^ conidia/mL. Before inoculation, the suspension was mixed to ensure uniform distribution of conidia. This experiment was designed to compare isolate aggressiveness after standardized wound-assisted inoculation and was not designed to test the ability of conidia to penetrate an intact leaf surface. Soybean leaves were wounded with an entomological needle midway between a vein and the leaf margin, and 20 µL of conidial suspension was applied to the wound. The same inoculum concentration and inoculation method were used for all isolates. Control leaflets were wounded using the identical entomological-needle procedure and received the same volume (20 µL) of sterile water without conidia. After inoculation, the plants were covered with polyethylene bags to provide high humidity, and the bags were removed after 3 days of incubation. Following inoculation, all treatments were maintained under identical growing conditions until the end of the experiment. Six individual soybean plants were used for each isolate. On each plant, the first and second trifoliate leaves above the pair of unifoliate leaves, counted from the bottom, were inoculated. For quantitative assessment of disease, one leaflet from the first trifoliate leaf of each plant was evaluated. Thus, six values of diseased area were obtained for each isolate, one from each plant. An individual plant was considered the experimental unit (*n* = 6). The negative control comprised six plants treated with sterile water instead of the conidial suspension; inoculation and selection of the leaflet for assessment followed the same procedure.

Disease area was assessed 7 days after inoculation. One assessed leaflet from the first trifoliate leaf of each plant was photographed separately. Images were analyzed using the LeafDoctor application installed on an iPhone SE 2 according to the developers’ recommendations [57]. Necrotic regions of the leaf tissue were delineated in each image. Chlorotic areas without visible necrosis were excluded from the measured area. The segmentation threshold was adjusted individually for each image using the same criterion for identifying necrotic tissue across all experimental treatments. The diseased area was expressed as a percentage of the total area of the analyzed leaflet. The image background and petiole were excluded from the analyzed area. Superficial mycelium was not considered diseased leaf tissue. One value from each plant was included in the statistical analysis; six observations were obtained for each treatment (*n* = 6). No visible disease symptoms were observed in the control treatment.

#### 4.5.3. Reisolation of Fungi from Inoculated Tissues

After completion of the artificial inoculation experiments, fungi were reisolated from soybean tissues that had developed symptoms to confirm the association between the observed lesions and the *Epicoccum* isolates used for inoculation. Tissue sterilization and mycelial cultivation were performed according to the procedure described in Section 4.1. Reisolated cultures were compared with the original cultures on the basis of cultural and morphological characteristics. For molecular comparison, the rDNA-ITS region was amplified and sequenced using the method described in Section 4.2. The resulting sequences were compared with the corresponding sequences of the original *Epicoccum* isolates. Control samples were processed using the same procedure. The results of reisolation, comparison of cultural and morphological characteristics, and ITS analysis were used as additional evidence that the inoculated *Epicoccum* cultures were associated with the lesions that developed under the corresponding experimental inoculation conditions; they were not used as evidence of natural infection.

### 4.6. Effects of Artificial Inoculation on Seed Germination and Colonization

The general procedure for artificial inoculation and subsequent germination of soybean seeds was based on approaches used to study seed pathogenicity of *Epicoccum* [12]. Visually healthy seeds of soybean cultivar Pripyat harvested in 2025 and showing no visible disease symptoms were used in the experiment. All 15 *Epicoccum* isolates and a negative control were included in the study. Before inoculation, three separate lots of 100 seeds were prepared for each treatment. Each lot was treated and assessed separately; a lot of 100 seeds was considered the experimental unit (*n* = 3).

Mycelium from 7-day-old *Epicoccum* cultures grown on PDA was used to prepare the inoculum. Mycelium was collected from the medium surface with a sterile scraper, transferred to sterile water, and washed three times with sterile water. The washed mycelium was homogenized aseptically by grinding in a sterile ceramic mortar with a sterile pestle. The concentration of mycelial fragments was determined by direct counting in a hemocytometer and adjusted with sterile water to 1 × 10^6^ fragments/mL. The inoculum was standardized according to the number of counted mycelial fragments. The viability of individual fragments and the fungal dry biomass were not independently quantified; therefore, equal fragment counts should not be interpreted as evidence of equal viable inoculum or equal biomass among isolates.

Before inoculation, seeds were surface-sterilized with 75% ethanol for 30 s, rinsed three times with sterile water, and dried in a laminar-flow cabinet. Each 100-seed lot was completely immersed in 200 mL of the mycelial suspension of the corresponding isolate for 1 h in a separate container. Control seed lots were separately immersed in sterile water for the same period. After treatment, the seeds from each lot were distributed among 10 Petri dishes, with 10 seeds per dish, on sterile filter paper moistened with sterile water. The dishes were incubated in sealed zip-lock bags at 25 °C in a KB 23 incubator. The dishes belonging to each replicate were placed in a separate zip-lock bag.

Germination energy was determined on day 5, and final germination was determined on day 8 after the start of germination, in accordance with the assessment times for soybean seeds [58]. When germination was assessed, seedlings with normally developed essential structures and without damage that would prevent further development were considered normal [58].

Germination energy (E) and germination (G) were calculated using the following equations:

*E* = *n*_5/_*N* × 100, (2)

*G* = *n*_8/_*N* × 100, (3)

where *N* is the number of seeds in a replicate, and n_5_ and n_8_ are the numbers of normally germinated seeds on days 5 and 8, respectively.

Visible seed surface colonization was assessed on day 8 simultaneously with the final germination assessment. Seeds with visually detectable fungal colonization of the surface, manifested by mycelial growth and/or sporulation, were considered affected regardless of whether they had germinated. Visible seed surface colonization (*C*) was calculated using the following equation:

*C* = *n*_c/_*N* × 100 (4)

where *n*_c_ is the number of seeds with visual signs of fungal colonization. The parameter “visible seed surface colonization” characterized external, visually detectable mycelial growth and/or sporulation and was not used as evidence of internal seed infection or of natural seedborne transmission. For each replicate, the parameters were calculated from the total number of corresponding seeds across all dishes belonging to that replicate, using 100 seeds as the denominator. Results were expressed as the mean ± standard deviation of the three replicate values. For correlation analysis, the reduction in germination was calculated as *L* = 100 − *G*; because germination in the control was 100%, this value was equivalent to the difference between the control and the treatment in percentage points. At the end of the experiment, fungi were reisolated from seedling tissues in treatments with 14 isolates and from nongerminated seeds in the treatment with isolate 152H. The resulting cultures were compared with the original cultures using the procedure described in Section 4.5.3.

### 4.7. Assessment of Extracellular Enzymatic Activity

#### 4.7.1. Preparation of Cultures and Test Media

Amylolytic, pectinolytic, cellulolytic, and proteolytic activities of all 15 *Epicoccum* isolates were assessed using semiquantitative substrate agar assays based on formation of a visually distinguishable substrate-hydrolysis zone in the medium [23,59]. A similar set of extracellular hydrolytic enzymes has previously been used for phenotypic characterization of *Epicoccum* species [6].

Before the assays, isolates were grown on PDA for 5–7 days at 25 °C. A 3-mm agar plug was cut with a sterile cork borer from the actively growing colony margin and placed mycelium-side down in the center of the corresponding test medium. Three independent Petri dishes were used for each isolate × enzymatic assay combination (*n* = 3).

A common mineral agar base of the following composition, g/L, was used for the amylolytic, pectinolytic, and cellulolytic assays: NaNO_3_, 2.0; KH_2_PO_4_, 1.0; MgSO_4_·7H_2_O, 0.5; KCl, 0.5; agar, 20.0; distilled water to 1 L. The specific substrate for the corresponding enzymatic assay was added to the base at the concentration indicated below. Before sterilization, the medium pH was adjusted to 5.6 with NaOH or HCl solutions using a pH meter. The media were sterilized under the conditions described in Section 4.4.1. After inoculation, the plates were incubated at 25 °C. Within each assay, all study isolates and the corresponding positive-control cultures were grown on medium from the same batch and assessed at the same time.

#### 4.7.2. Amylolytic Activity

Amylolytic activity was determined using the method of Hankin and Anagnostakis with modifications on a mineral medium containing 5.0 g/L soluble potato starch (analytical grade; Khimpribor-SPb, St. Petersburg, Russia) [59]. After 5 days of incubation, the entire surface of the medium was covered with Lugol’s solution for 1–2 min, after which excess solution was removed. Nonhydrolyzed starch stained dark, whereas a light zone in the medium corresponded to the area of starch hydrolysis.

#### 4.7.3. Pectinolytic Activity

Pectinolytic activity was determined on the mineral base containing 10.0 g/L DIPECTA AG366 pectin (catalog no. ACC 00710; Agdia, Inc., Elkhart, IN, USA). The method is based on precipitation of nonhydrolyzed acidic polysaccharides and is widely used to detect pectinolytic activity in fungi [60]. After 5 days of incubation, the surface of the medium was flooded with 1% cetyltrimethylammonium bromide (CTAB) solution and incubated for 15–20 min. After removal of CTAB, a clear zone in the medium corresponding to the area of pectic-substrate hydrolysis was recorded.

#### 4.7.4. Cellulolytic Activity

Cellulolytic activity was determined on the mineral base containing 10.0 g/L carboxymethyl cellulose (CMC), with modifications to published methods [61,62]. After 5 days of incubation, the medium was flooded with 0.1% Congo red solution for 15–20 min, after which the dye was removed and the medium was treated with 1 M NaCl for 10–15 min. A light area in the medium against the stained background was recorded as the CMC hydrolysis zone.

#### 4.7.5. Proteolytic Activity

Proteolytic activity was determined, with modifications, on milk agar of the following composition, g/L: skim milk powder, 10.0; NaCl, 5.0; agar, 18.0; distilled water to 1 L [59,63]. The medium components were prepared separately. Skim milk powder was dissolved in half of the final volume of distilled water and subjected to three brief heating cycles to boiling, with 5 min of cooling between cycles; the milk component was not autoclaved to avoid protein coagulation. Agar and NaCl were dissolved in the remaining half of the water volume and sterilized by autoclaving. After the agar base had cooled, the two components were aseptically combined in a laminar-flow cabinet, mixed, and dispensed into sterile Petri dishes. After 5 days of cultivation, a clear area in the medium resulting from hydrolysis of milk protein was recorded. No additional visualization treatment was performed. The result was interpreted as total extracellular proteolytic activity without assigning the reaction to a specific class of proteases.

#### 4.7.6. Positive Controls and Calculation of Relative Enzymatic Activity

Positive-control cultures were used to verify the performance of the assay systems. *Fusarium oxysporum* MP-1 was used in all four assays; additionally, *Aspergillus niger* RT598 was used in the amylolytic assay, *Trichoderma harzianum* Th2RI99 in the cellulolytic assay, *Bacillus amyloliquefaciens* MBI 600 in the proteolytic assay, and *Fusarium graminearum* PH-1 in the pectinolytic assay. Information on the positive-control cultures used, their purpose, and their REA values is provided in Table S9.

On day 5 of cultivation, colony diameter (D_c_) was measured in two mutually perpendicular directions with an ADA Mechanic 150 PRO digital caliper (ADA Instruments, Shenzhen, China), and the values were averaged. After visualization of the corresponding substrate assay, the full diameter of the visible hydrolysis area (D_h_) was determined in the same manner along its outer boundaries through the center of the colony. For the proteolytic assay, which required no additional visualization treatment, both parameters were recorded directly on day 5. The hydrolysis area was assessed from the bottom of the Petri dish. When D_h_ was measured, the visually distinguishable area of substrate hydrolysis, including the portion beneath the colony, was included. Relative enzymatic activity (REA) was calculated using the following equation [59,62]:

REA = D_h/_D_c_, (5)

where D_h_ is the mean full diameter of the visible hydrolysis area, mm, and D_c_ is the mean colony diameter, mm. When no visually distinguishable substrate-hydrolysis area was present, REA = 0 was recorded. Values of 0 < REA < 1 corresponded to a hydrolysis area with a smaller diameter than the colony. This parameter was used exclusively as a semiquantitative index of relative extracellular enzymatic activity and was not interpreted as absolute catalytic enzyme activity.

For each isolate, the mean REA ± standard deviation was calculated from three experimental replicates (*n* = 3). Positive-control cultures were used to verify the performance of the corresponding assay systems and were not included in statistical comparisons among *Epicoccum* isolates. Proteolytic activity was not statistically compared among isolates because no visible hydrolysis zone was present in any of the three replicates for any study isolate, and REA was therefore set to 0.

### 4.8. Assessment of Sensitivity to Azoxystrobin, Boscalid, Fludioxonil, and Tebuconazole Using EC_50_

Fungicide sensitivity of the isolates was assessed with modifications to a previously published method [26]. Effective concentrations (EC_50_) at which mycelial growth was inhibited by 50% relative to the control without fungicide were determined.

Technical-grade active ingredients (95% active ingredient) were used: azoxystrobin (strobilurin, inhibitor of complex III of the mitochondrial respiratory chain [QoI], FRAC 11; manufacturer: Haili Guixi Chemical Co. Ltd., Yingtan, China), boscalid (succinate dehydrogenase inhibitor, SDHI, FRAC 7; manufacturer: Haili Guixi Chemical Co. Ltd., Yingtan, China), fludioxonil (phenylpyrrole, inhibitor of osmotic signal transduction [MAP/histidine kinase], FRAC 12; manufacturer: Hubei Kang Bao Tai Fine-Chemical Co. Ltd., Hubei, China), and tebuconazole (triazole, sterol demethylation inhibitor, DMI, FRAC 3; manufacturer: Hubei Kang Bao Tai Fine-Chemical Co. Ltd., Hubei, China).

Before addition to the medium, azoxystrobin, boscalid, fludioxonil, and tebuconazole were dissolved in 99.8% dimethyl sulfoxide (DMSO; Komponent-Reaktiv LLC, Moscow, Russia). A separate stock solution was prepared for each active ingredient, and the working solutions covering the required concentration range were obtained from it by serial dilution in DMSO. Stock- and working-solution concentrations, as well as the final concentrations in the medium, were calculated taking into account the 95% active-ingredient content of the compounds. For each active ingredient, a constant volume of the corresponding working solution was added to the molten medium, so that the final DMSO content was identical in all plates of a given series and in the corresponding fungicide-free control. PDA containing DMSO served as the control for all active ingredients.

The prepared solutions were added with automatic pipettes to sterile glass bottles containing previously autoclaved PDA cooled to 47 °C in a water bath, gently mixed to avoid bubble formation, and dispensed into sterile Petri dishes (20 mL per dish) using a Levo Plus electronic dispenser (DLAB Scientific Co., Ltd., Beijing, China) in a sterile laminar-flow cabinet. Final concentrations in PDA were as follows: boscalid, 0 (PDA + DMSO), 0.01, 0.1, and 1 µg/mL; fludioxonil, 0 (PDA + DMSO), 0.01, 0.1, and 1 µg/mL; and tebuconazole, 0 (PDA + DMSO), 50, 100, and 150 µg/mL. For tebuconazole, the estimated EC50 values (3.735-49.542 µg/mL) were below or close to the lowest tested concentration (50 µg/mL); they were therefore obtained by extrapolation of the fitted 4PL curves below the tested concentration range and should be regarded as approximate estimates suitable only for relative comparison of isolates tested under identical conditions.

For azoxystrobin, preliminary screening of 14 isolates was performed at concentrations of 0, 800, 900, and 1000 µg/mL. Because 50% growth inhibition was not achieved for these isolates even at the highest tested concentration of 1000 µg/mL, exact EC_50_ values beyond the tested range were not extrapolated and were recorded as EC_50_ > 1000 µg/mL. For the *E. tobaicum* isolate 152H, which showed markedly greater sensitivity to azoxystrobin, an additional assay was performed at concentrations of 0, 0.01, 0.1, and 1 µg/mL, allowing EC_50_ to be determined within the experimental range. Reporting values beyond the upper limit of the tested range in this way is preferable to assigning artificially calculated values because it avoids unjustified extrapolation of the dose-response model [26,64]. Salicylhydroxamic acid (SHAM) was not used in the present experiment; therefore, the azoxystrobin EC_50_ values characterized the phenotypic response of mycelial growth under the conditions of this in vitro assay and were not used alone as evidence of a resistance mechanism.

The plates were left for 30 min in an operating laminar-flow cabinet to allow the medium to solidify. Agar plugs (5 mm) were cut with a laboratory cork borer from the periphery of a five-day-old colony of each isolate and placed with sterile metal forceps in the center of each PDA plate containing the corresponding fungicide concentration.

Colony diameters were measured in two mutually perpendicular directions on day 14 of incubation at 25 °C in the dark, as described above. Three Petri dishes were used in a single experimental series for each isolate × fungicide concentration combination, including the corresponding fungicide-free control. Three independent dose-response curves were fitted for each isolate, yielding three EC_50_ estimates (*n* = 3). The two mutually perpendicular measurements of the diameter of a single colony were averaged to obtain one value per plate. EC_50_ values were calculated by nonlinear regression in GraphPad Prism 9.2.0 (GraphPad Software Inc., Boston, MA, USA) using a four-parameter logistic model with a variable slope (4PL, variable slope) describing the relationship between colony growth and fungicide concentration [64].

### 4.9. Statistical Analysis and Data Presentation

Throughout this study, the following parameters are distinguished and are not used interchangeably: necrotic lesion diameter (mycelial-plug leaf assay, mm); proportion of necrotic leaflet area (wound-assisted conidial leaf assay, %); germination energy (normally germinated seeds on day 5, %); final germination (normally germinated seeds on day 8, %); and visible seed surface colonization (seeds with externally visible mycelial growth and/or sporulation on day 8, %).

Analysis of variance and Duncan’s multiple comparisons were performed in Statistica 12.0 (TIBCO Software Inc., Palo Alto, CA, USA), whereas graphs and concentration-response curves were generated in GraphPad Prism 9.2.0 (GraphPad Software, Boston, MA, USA). Quantitative data were expressed as the arithmetic mean ± standard deviation (SD). For EC_50_, the mean and SD were calculated from three independently estimated EC_50_ values. Differences were considered statistically significant at *p* < 0.05. For each one-way ANOVA, the effect size was calculated as η^2^ = (F × df__between_)/(F × df__between_ + df__error_) and is reported in Table S13. For the leaf and seed assays, 95% confidence intervals of the treatment means were calculated as mean ± t(0.975, n−1) × SD/√n and are reported in Tables S6–S8; for treatments in which all replicates gave identical values, the variance estimate is zero and a t-based confidence interval is not informative, and these cases are indicated accordingly.

Differences in the rate of increase in colony diameter among the 15 isolates were analyzed separately for each culture medium (PDA, MEA, and OA) using one-way analysis of variance (ANOVA). For each isolate on each medium, values from three separate Petri dishes were used (*n* = 3). Multiple comparisons were performed using Duncan’s multiple range test at α = 0.05 [65]. The letter groups in Table S5 characterize differences among isolates only within a given culture medium and are not intended for comparisons among media.

The remaining experimental variables were analyzed separately using one-way ANOVA followed by Duncan’s multiple range test at α = 0.05. Experimental units were defined before analysis, and subsamples or repeated measurements within an experimental unit were not treated as independent replicates. In the mycelial-plug inoculation experiment, the 15 *Epicoccum* isolates and the negative control were compared. Necrotic lesion diameter measurements from individual plants were analyzed, with one value recorded per plant; eight plants were assessed per treatment (*n* = 8), except for isolates 133H and 143H, for which nine plants were assessed (*n* = 9). Each value represented the mean of two mutually perpendicular measurements of a single necrotic lesion, and the two measurements of one lesion were not treated as separate replicates. Because the design was slightly unbalanced, Duncan’s multiple range test was applied using the harmonic mean of the two group sizes being compared. In the wound-assisted conidial-suspension experiment, nine *E. tobaicum* isolates and the negative control were compared. Six plants were included in each treatment, and one value representing the proportion of necrotic leaflet area was recorded for each plant. Thus, the plant served as the experimental unit (*n* = 6). Necrotic lesion diameter was analyzed without transformation; percentage values of leaf disease in the conidial-suspension assay were arcsine-transformed before analysis.

In the seed experiment, a separately treated lot of 100 seeds was the biological experimental unit. For each of the 15 isolates and the negative control, three independently treated lots were analyzed (*n* = 3); the individual seeds and the 10 Petri dishes belonging to a lot were subsamples and were not treated as independent replicates. Percentage data from the seed experiment were transformed as arcsin√(*p*/100) before ANOVA. Compliance with the ANOVA assumptions was assessed for each endpoint of this experiment. Normality of the residuals was evaluated using the Shapiro-Wilk test, and homogeneity of variance was evaluated using Levene’s test based on absolute deviations from the group median (Brown-Forsythe modification), which is robust to treatments showing zero within-treatment variance. For germination energy and final germination, both assumptions were satisfied (Shapiro-Wilk W = 0.959, *p* = 0.095 and W = 0.980, *p* = 0.559; Brown-Forsythe *p* = 0.613 and *p* = 0.894, respectively). For visible seed surface colonization, neither assumption was satisfied (Shapiro-Wilk W = 0.544, *p* < 0.001; Brown-Forsythe *p* = 0.014), because 13 of the 16 treatments gave identical values in all three replicate lots and therefore had zero within-treatment variance. A rank-based alternative was examined for this endpoint but proved uninformative: owing to the large number of ties at 100%, the Kruskal-Wallis test followed by Dunn’s test with Benjamini-Hochberg correction did not separate the uninoculated control (0% colonization) from treatments showing 84–96% colonization (adjusted *p* > 0.5). The parametric framework was therefore retained for all three endpoints, and the grouping letters for visible seed surface colonization are interpreted descriptively rather than as formal pairwise inference.

Amylolytic, pectinolytic, and cellulolytic activities were analyzed separately for each enzymatic assay using one-way ANOVA followed by Duncan’s test. Three replicates were used for each isolate (*n* = 3). REA values were analyzed without transformation. Positive-control cultures were used to verify the performance of the assay systems and were not included in statistical comparisons among isolates. Proteolytic activity was not analyzed statistically because the trait showed no variability: REA = 0 in all replicates for all study isolates.

For assessment of sensitivity to boscalid, fludioxonil, and tebuconazole, three independently calculated EC_50_ values were used for each isolate (*n* = 3). Before ANOVA, EC_50_ values were transformed as log10(EC_50_); untransformed EC_50_ values in µg/mL were presented in tables and figures as the mean ± SD. EC_50_ values were calculated in GraphPad Prism 9.2.0 by nonlinear regression using a four-parameter logistic model with a variable slope (4PL, variable slope), as described in Section 4.8. Azoxystrobin EC_50_ values > 1000 µg/mL were treated as right-censored and were not included in ANOVA or multiple comparisons.

Results of Duncan’s test were denoted by lowercase Latin letters. Within a given parameter, treatments sharing no common letter were considered statistically different at *p* < 0.05; sharing a common letter indicated that no difference was detected by this test and was not interpreted as evidence of statistical equivalence.

Correlation analysis was performed in R version 4.5.2 using RStudio version 2026.01.1+403. Associations among phenotypic traits were assessed using Spearman’s rank correlation coefficient, with one mean value of each trait for each of the 15 isolates (*n* = 15) [66]. Six traits were included in the analysis: necrotic lesion diameter after inoculation with mycelial plugs, reduction in seed germination, mean rate of increase in colony diameter across the three media, and amylolytic, pectinolytic, and cellulolytic activities. For each isolate, the mean rate of increase in colony diameter across the three media was calculated as the arithmetic mean of the corresponding mean values on PDA, MEA, and OA. Reduction in seed germination was defined as 100 − G, where G denotes final germination expressed as a percentage. The conidial leaf-assay variable was not included because it was obtained only for nine *E. tobaicum* isolates; proteolytic activity was excluded because of the absence of variability. Control treatments were not included in the correlation analysis. A total of 15 pairwise correlations were calculated for the six traits. The *p*-values for all 15 comparisons were adjusted jointly using the Benjamini-Hochberg procedure to control the false discovery rate (FDR) [67]. Correlations were considered statistically significant at an adjusted *p*-value < 0.05. For each pair of traits, Spearman’s ρ, the raw *p*-value, and the adjusted *p*-value were reported. A separate statistical effect of species identity was not assessed because species were unevenly represented in the collection; the results of analysis of variance and multiple comparisons were interpreted at the level of individual isolates.

## 5. Conclusions

To our knowledge, this study provides the first molecularly confirmed records of *E. tobaicum*, *E. mezzettii*, and *E. oryzae* associated with soybean in Russia. Controlled inoculation assays provided evidence of the leaf-damaging capacity and pathogenic potential of individual *E. tobaicum* isolates; because the conidial assay was performed on standardized needle wounds, it does not establish the ability of these fungi to initiate infection on intact leaves. Artificial seed inoculation showed that individual *E. tobaicum* and *E. mezzettii* isolates can reduce germination under the experimental conditions, although the differences among isolates may partly reflect inoculum characteristics, because the viability and biomass of the mycelial-fragment suspensions were not independently quantified. Despite marked phenotypic variability among the isolates, the five deliberately selected genomes had predicted functional repertoires of similar size, and the isolate-associated genomic differences identified require functional validation. Boscalid and fludioxonil exhibited relatively low EC_50_ values in vitro, whereas most isolates showed low apparent sensitivity to azoxystrobin in the mycelial-growth assay; these data do not establish field resistance or product efficacy. The pathogenicity of these fungi on unwounded plants, their natural seedborne transmission, the functional roles of the candidate genomic features, and the efficacy of the fungicides under field conditions require further investigation.

## Figures and Tables

**Table 1:** Origin, species identity, and nucleotide sequence accession numbers of the studied *Epicoccum* isolates obtained from soybean.

Isolate ID	Species	Region	District/Municipal District	Isolation Source	Year of Isolation	GenBank Accession Numbers		
						rDNA-ITS	*RPB2*	*TUB2*
164H	*Epicoccum oryzae*	Khabarovsk region	Vyazemsky District	Seeds	2024	PZ830776	PZ851350	PZ844405
197H	*Epicoccum mezzettii*	Amur region	Tambovsky District	Seeds	2026	PZ830779	PZ851353	PZ844408
250H	*Epicoccum mezzettii*	Jewish Autonomous region	Birobidzhansky District	Seeds	2025	PZ830781	PZ851355	PZ844410
159H	*Epicoccum mezzettii*	Khabarovsk region	Vyazemsky District	Leaves	2024	PZ830775	PZ851349	PZ844404
190H	*Epicoccum mezzettii*	Khabarovsk region	Khabarovsky District	Leaves	2024	PZ830777	PZ851351	PZ844406
46H	*Epicoccum mezzettii*	Khabarovsk region	Vaninsky District	Leaves	2025	PZ830769	PZ851343	PZ844398
193H	*Epicoccum tobaicum*	Khabarovsk region	Khabarovsky District	Leaves	2025	PZ830778	PZ851352	PZ844407
152H	*Epicoccum tobaicum*	Khabarovsk region	Khabarovsky District	Leaves	2025	PZ830774	PZ851348	PZ844403
288H	*Epicoccum tobaicum*	Jewish Autonomous region	Birobidzhansky District	Leaves	2024	PZ830782	PZ851356	PZ844411
17H	*Epicoccum tobaicum*	Tula region	Yefremov Urban Okrug	Leaves	2024	PZ830768	PZ851342	PZ844397
124H	*Epicoccum tobaicum*	Khabarovsk region	Khabarovsky District	Leaves	2024	PZ830771	PZ851345	PZ844400
143H	*Epicoccum tobaicum*	Khabarovsk region	Lazo District	Leaves	2024	PZ830773	PZ851347	PZ844402
200H	*Epicoccum tobaicum*	Jewish Autonomous region	Birobidzhansky District	Seeds	2024	PZ830780	PZ851354	PZ844409
120H	*Epicoccum tobaicum*	Khabarovsk region	Bikinsky District	Leaves	2024	PZ830770	PZ851344	PZ844399
133H	*Epicoccum tobaicum*	Khabarovsk region	Ulchsky District	Seeds	2024	PZ830772	PZ851346	PZ844401

## Data Availability

The ITS, *RPB2*, and *TUB2* sequences generated in this study have been deposited in GenBank; individual accession numbers are provided in Supplementary Table S1 (ITS: PZ830768-PZ830782; *RPB2*: PZ851342-PZ851356; *TUB2*: PZ844397-PZ844411). Raw paired-end whole-genome sequencing (WGS) reads are available in the NCBI Sequence Read Archive under BioProject PRJNA1521810; individual BioSample and SRA Run accession numbers are provided in Supplementary Table S2. The corresponding genome assemblies have been submitted to NCBI GenBank under the same BioProject; their WGS/GenBank accession numbers had not been assigned at the time of submission of this revised manuscript and will be provided to the editorial office before publication.

## Supplementary Materials

The following supporting information can be downloaded at https://www.mdpi.com/article/10.3390/plants15213388/s1: Supplementary Figure S1: Representative inhibition of mycelial growth of *Epicoccum* isolates by azoxystrobin, boscalid, fludioxonil, and tebuconazole in vitro; Supplementary Table S1: Sequences and geographic origin of isolates used in the multilocus phylogenetic analysis of *Epicoccum*; Supplementary Table S2: Whole-genome sequencing, assembly, annotation, read-mapping and BUSCO statistics; Supplementary Table S3: Comparative genomic characteristics of the five sequenced *Epicoccum* isolates; Supplementary Table S4: Validation of selected orthogroup copy-number and presence/absence candidates; Supplementary Table S5: Mean rate of increase in colony diameter of the studied *Epicoccum* isolates on PDA, MEA, and OA; Supplementary Table S6: Aggressiveness of the studied *Epicoccum* isolates on soybean leaves following inoculation with mycelial plugs; Supplementary Table S7: Aggressiveness of *Epicoccum tobaicum* isolates on soybean leaves following inoculation with a conidial suspension; Supplementary Table S8: Germination energy, final germination, and visible seed surface colonization of soybean seeds following artificial inoculation with *Epicoccum* isolates; Supplementary Table S9: Positive-control cultures and their activity in substrate agar assays; Supplementary Table S10: Relative extracellular enzymatic activity of the studied *Epicoccum* isolates in substrate agar assays; Supplementary Table S11: Spearman rank correlations among aggressiveness, reduction in seed germination, rate of increase in colony diameter, and extracellular enzymatic activity of *Epicoccum* isolates; Supplementary Table S12: In vitro sensitivity of the studied *Epicoccum* isolates to azoxystrobin, boscalid, fludioxonil, and tebuconazole; Supplementary Table S13: Summary results of one-way analysis of variance for quantitative traits of the studied *Epicoccum* isolates.

## Abbreviations

The following abbreviations are used in this manuscript:

ANOVA	Analysis of variance
BGC	Biosynthetic gene cluster
BUSCO	Benchmarking Universal Single-Copy Orthologs
CAZymes	Carbohydrate-active enzymes
EC_50_	Effective concentration causing 50% inhibition of mycelial growth
FDR	False discovery rate
ITS	Internal transcribed spacer
MEA	Malt extract agar
OA	Oatmeal agar
PDA	Potato dextrose agar
REA	Relative enzymatic activity
*RPB2*	Gene encoding the second-largest subunit of RNA polymerase II
SD	Standard deviation
SHAM	Salicylhydroxamic acid
*TUB2*	β-Tubulin gene
