# Supplementary material for: Epicoccum Associated with Soybean in Russia: Species Diversity, Pathogenic Potential, and Genomic Features
Source: Plants (Basel). 2026 Nov 1;15(21):3388. doi: 10.3390/plants15213388 (PMC13766208; doi:10.3390/plants15213388)
Supplement: Supplementary file 1 [file plants-15-03388-s001.zip › Supplementary Tables S5-S13; Figure S1.pdf]

plants

MDPI

Article

Epicoccum Associated with Soybean in Russia: Species Diversity, Pathogenic Potential, and Genomic Features

Supplementary materials

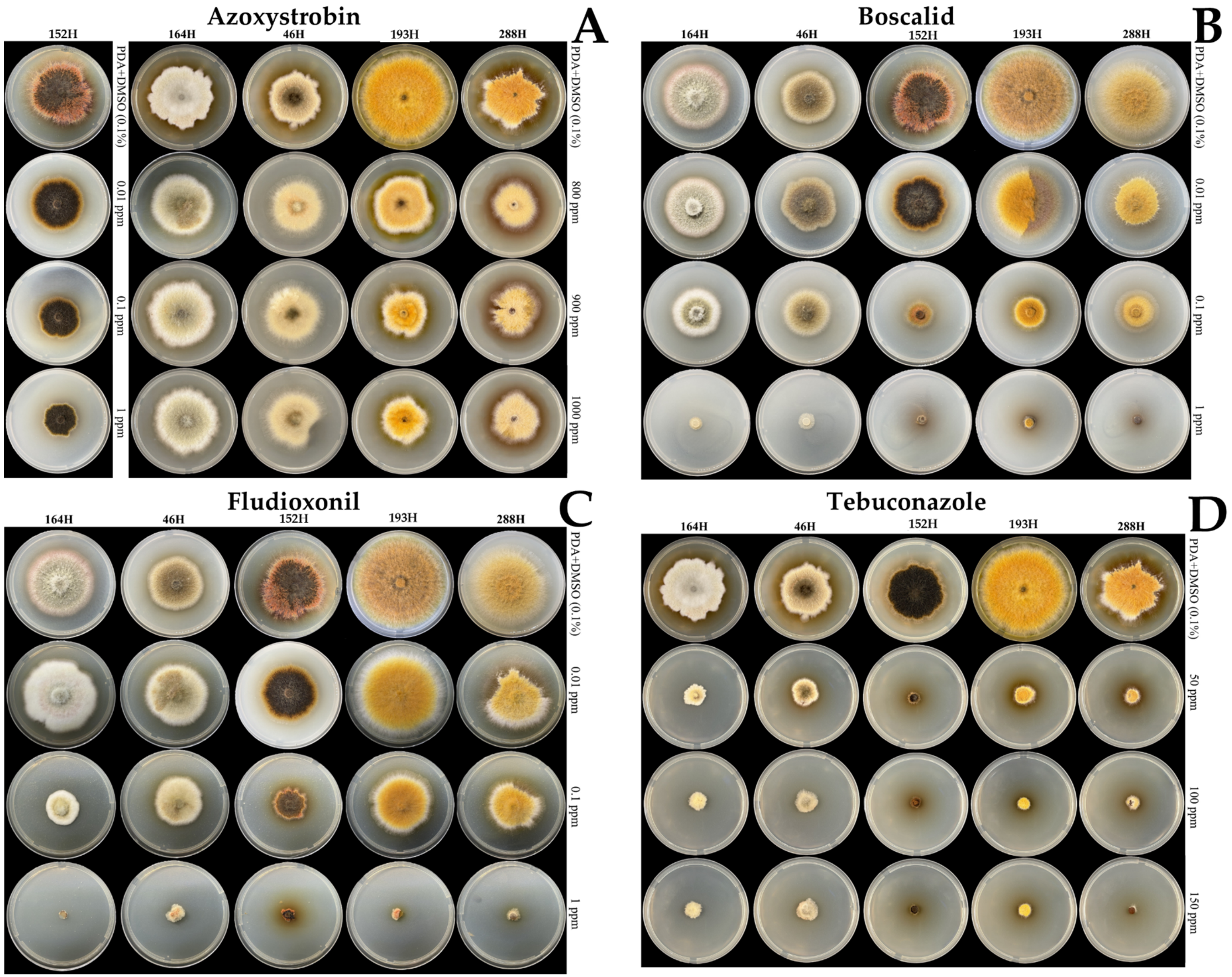

Supplementary Figure S1. Representative inhibition of mycelial growth of *Epicoccum* isolates by azoxystrobin, boscalid, fludioxonil, and tebuconazole *in vitro*. (A) Azoxystrobin; (B) boscalid; (C) fludioxonil; (D) tebuconazole. Representative isolates 164H, 46H, 152H, 193H, and 288H are shown on PDA containing the corresponding concentrations of the active ingredients. Control media contained no fungicide: PDA with DMSO was used for azoxystrobin, boscalid, fludioxonil, and

tebuconazole. The final solvent content in the corresponding control was identical to that in the experimental treatment being compared. Concentrations on the panels are given in ppm (1 ppm = 1 μg/mL).

Supplementary Table S5. Mean rate of increase in colony diameter of the studied *Epicoccum* isolates on PDA, MEA, and OA

Isolate	Species	PDA		MEA		OA	
		Rate of increase in diameter, mm/day, mean ± SD	Duncan multiple range test (grouping letters)	Rate of increase in diameter, mm/day, mean ± SD	Duncan multiple range test (grouping letters)	Rate of increase in diameter, mm/day, mean ± SD	Duncan multiple range test (grouping letters)
164H	*E. oryzae*	6.810 ± 0.082	de	4.524 ± 0.218	e	5.095 ± 0.165	c
197H	*E. mezzettii*	2.905 ± 0.082	g	2.881 ± 0.230	h	2.786 ± 0.214	e
250H	*E. mezzettii*	3.286 ± 0.143	g	2.857 ± 0.143	h	3.310 ± 0.289	d
159H	*E. mezzettii*	5.286 ± 0.143	f	3.929 ± 0.189	f	2.738 ± 0.289	e
190H	*E. mezzettii*	5.262 ± 0.694	f	3.238 ± 0.218	gh	3.214 ± 0.214	d
46H	*E. mezzettii*	2.810 ± 0.541	g	3.381 ± 0.165	g	2.571 ± 0.286	e
193H	*E. tobaicum*	10.000 ± 0.143	a	5.214 ± 0.071	d	6.429 ± 0.143	b
152H	*E. tobaicum*	3.190 ± 0.082	g	3.381 ± 0.297	g	5.048 ± 0.218	c
288H	*E. tobaicum*	6.524 ± 0.436	e	5.810 ± 0.360	c	7.190 ± 0.165	a
17H	*E. tobaicum*	6.714 ± 0.515	de	7.381 ± 0.218	b	6.476 ± 0.218	b
124H	*E. tobaicum*	7.381 ± 0.218	d	7.381 ± 0.082	b	6.286 ± 0.143	b
143H	*E. tobaicum*	8.833 ± 0.352	bc	5.476 ± 0.218	cd	6.619 ± 0.218	b
200H	*E. tobaicum*	8.571 ± 0.495	c	5.524 ± 0.297	cd	5.071 ± 0.214	c
120H	*E. tobaicum*	6.333 ± 0.861	e	5.857 ± 0.143	c	6.571 ± 0.143	b
133H	*E. tobaicum*	9.333 ± 0.297	ab	8.143 ± 0.286	a	6.667 ± 0.218	b

Note. Values are presented as the mean ± standard deviation (n = 3). For each culture medium, differences among isolates were evaluated using one-way analysis of variance (ANOVA) followed by Duncan's multiple range test. Different lowercase letters within a given culture medium indicate statistically significant differences among isolates at $p < 0.05$. Letter designations are not comparable among different culture media.

Supplementary Table S6. Aggressiveness of the studied *Epicoccum* isolates on soybean leaves following inoculation with mycelial plugs.

Isolate	Species	Necrotic lesion diameter, mm	SD	95% CI (mm)	n, plants	Duncan multiple range test (grouping letters)
164H	*E. oryzae*	2.50	1.34	1.38-3.62	8	cde
197H	*E. mezzettii*	2.81	0.70	2.22-3.40	8	cde
250H	*E. mezzettii*	3.62	0.99	2.80-4.45	8	cd
159H	*E. mezzettii*	2.88	1.09	1.96-3.79	8	cd
190H	*E. mezzettii*	2.06	0.56	1.59-2.53	8	de
46H	*E. mezzettii*	2.69	1.49	1.44-3.93	8	cde
193H	*E. tobaicum*	1.50	0.53	1.05-1.95	8	de
152H	*E. tobaicum*	3.88	2.26	1.98-5.77	8	cd
288H	*E. tobaicum*	11.06	4.32	7.45-14.68	8	a
17H	*E. tobaicum*	3.00	3.09	0.41-5.59	8	cd
124H	*E. tobaicum*	12.75	5.03	8.55-16.95	8	a
143H	*E. tobaicum*	6.83	2.02	5.28-8.38	9	b
200H	*E. tobaicum*	5.19	3.95	1.88-8.49	8	bc
120H	*E. tobaicum*	4.00	1.95	2.37-5.63	8	cd
133H	*E. tobaicum*	7.72	3.52	5.02-10.43	9	b
Control	-	0.00	0.00	-[a]	8	e

Note. [a] - All plants in the control showed no lesions (zero variance); the confidence interval is not applicable. Values are presented as the mean ± standard deviation for the individual plants assessed in each treatment: eight plants per treatment (n = 8), except for isolates 133H and 143H, for which nine plants were assessed (n = 9). One inoculated leaflet was assessed on each plant, and the necrotic lesion diameter for each plant was calculated as the mean of two mutually perpendicular measurements of a single necrotic lesion; the two measurements of one lesion were not treated as separate replicates. All available observations were included in the analysis. Because the design was slightly unbalanced, Duncan's multiple range test was applied using the harmonic mean of the two group sizes being compared. Confidence intervals were calculated as mean ± t(0.975, n-1) × SD/√n. Differences among treatments were evaluated using one-way analysis of variance (ANOVA) followed by Duncan's multiple range test. Different lowercase letters indicate statistically significant differences among treatments at $p < 0.05$. The control consisted of a sterile agar plug without fungal mycelium.

Supplementary Table S7. Aggressiveness of *Epicoccum tobaicum* isolates on soybean leaves following wound-assisted inoculation with a conidial suspension.

Isolate	Proportion of necrotic leaflet area, %	SD	95% CI	n, plants	Duncan multiple range test (grouping letters)
193H	2.42	0.74	1.64-3.20	6	e
152H	19.60	8.52	10.66-28.54	6	b
288H	26.17	8.93	16.79-35.54	6	a
17H	3.13	0.44	2.68-3.59	6	e
124H	3.32	0.66	2.62-4.01	6	e
143H	9.25	1.34	7.84-10.66	6	d
200H	8.87	4.47	4.17-13.56	6	cd
120H	3.78	1.11	2.62-4.95	6	ce
133H	9.63	4.30	5.12-14.15	6	d
Control	0.00	0.00	-[a]	6	e

Note. [a]-No lesions developed in the control (zero variance); the confidence interval is not applicable. Values are presented as the mean ± standard deviation for six plants in each treatment. On day 7 after inoculation, one leaflet from the first trifoliate leaf was assessed on each plant; one value per plant was included in the analysis (n = 6). The parameter represents the proportion of necrotic tissue relative to the total leaf-blade area; chlorotic areas without necrosis were excluded. Differences among treatments were evaluated using one-way analysis of variance (ANOVA) followed by Duncan's multiple range test. Different lowercase letters indicate statistically significant differences among treatments at $p < 0.05$. The control treatment was treated with sterile water without fungal conidia.

Supplementary Table S8. Germination energy, final germination, and visible seed surface colonization of soybean seeds following artificial inoculation with *Epicoccum* isolates

Isolate	Species	Germination energy			Final germination			Visible seed surface colonization		
		Mean germination energy, % ± SD	Duncan multiple range test (grouping letters)	95% CI (%)	Mean final germination, % ± SD	Duncan multiple range test (grouping letters)	95% CI (%)	Mean visible seed surface colonization, % ± SD	Duncan multiple range test (grouping letters)	95% CI (%)
164H	*E. oryzae*	84.3 ± 12.3	bcd	53.7-100.0	94.3 ± 3.8	bc	84.9-100.0	100.0 ± 0.0	a	-
197H	*E. mezzettii*	90.3 ± 4.5	ab	79.1-100.0	92.3 ± 3.8	bcd	82.9-100.0	100.0 ± 0.0	a	-
250H	*E. mezzettii*	87.0 ± 2.6	bcd	80.4-93.6	88.0 ± 7.2	cde	70.1-100.0	100.0 ± 0.0	a	-
159H	*E. mezzettii*	84.7 ± 3.5	bcde	75.9-93.4	94.7 ± 2.9	bc	87.5-100.0	100.0 ± 0.0	a	-
190H	*E. mezzettii*	60.0 ± 6.9	gh	42.8-77.2	66.7 ± 5.5	fg	53.0-80.3	100.0 ± 0.0	a	-
46H	*E. mezzettii*	92.3 ± 3.1	ab	84.7-99.9	94.3 ± 4.0	bc	84.3-100.0	100.0 ± 0.0	a	-
193H	*E. tobaicum*	54.3 ± 18.4	h	8.5-100.0	60.3 ± 2.5	g	54.1-66.6	92.0 ± 4.6	c	80.6-100.0
152H	*E. tobaicum*	0.0 ± 0.0	i	-	0.0 ± 0.0	h	-	100.0 ± 0.0	a	-
288H	*E. tobaicum*	76.7 ± 6.1	cdef	61.5-91.8	82.7 ± 10.4	de	56.8-100.0	96.0 ± 2.0	b	91.0-100.0

Isolate	Species	Germination energy			Final germination			Visible seed surface colonization		
		Mean germination energy, % ± SD	Duncan multiple range test (grouping letters)	95% CI (%)	Mean final germination, % ± SD	Duncan multiple range test (grouping letters)	95% CI (%)	Mean visible seed surface colonization, % ± SD	Duncan multiple range test (grouping letters)	95% CI (%)
17H	*E. tobaicum*	72.3 ± 5.1	efg	59.6-85.1	78.3 ± 11.2	ef	50.4-100.0	100.0 ± 0.0	a	-
124H	*E. tobaicum*	76.3 ± 4.6	cdef	64.9-87.8	84.7 ± 8.0	de	64.7-100.0	100.0 ± 0.0	a	-
143H	*E. tobaicum*	88.7 ± 3.8	abc	79.3-98.1	96.3 ± 1.2	bc	93.5-99.2	100.0 ± 0.0	a	-
200H	*E. tobaicum*	92.0 ± 2.0	ab	87.0-97.0	96.7 ± 2.1	b	91.5-100.0	100.0 ± 0.0	a	-
120H	*E. tobaicum*	74.0 ± 8.5	defg	52.8-95.2	90.3 ± 6.4	bcd	74.6-100.0	100.0 ± 0.0	a	-
133H	*E. tobaicum*	70.3 ± 5.1	fg	57.6-83.1	82.7 ± 5.1	de	69.9-95.4	84.3 ± 2.9	d	77.2-91.5
Control	-	96.3 ± 1.2	a	93.5-99.2	100.0 ± 0.0	a	-	0.0 ± 0.0	e	-

Note. CI - confidence interval. Values are presented as the mean ± standard deviation of three replicates (n = 3); each replicate represented a separately treated lot of 100 seeds. Before statistical analysis, percentage data were transformed using arcsin√(p/100). Differences among treatments were evaluated using one-way ANOVA followed by Duncan's multiple range test. Different lowercase letters within each parameter indicate statistically significant differences among treatments at $p < 0.05$; for visible seed surface colonization these letters are interpreted descriptively, because 13 of the 16 treatments showed zero within-treatment variance (see Section 4.9). Confidence intervals were calculated as mean ± t(0.975, n-1) × SD/√n; a dash indicates treatments in which all three replicate lots gave identical values, for which the variance estimate is zero and a t-based interval is not informative. The table presents untransformed percentage values.

Supplementary Table S9. Positive-control cultures and their activity in substrate agar assays.

Species	Strain	Enzymatic assay	REA ± SD	Culture source	Accession number and record role	Source / publication
Fusarium oxysporum	MP-1	Amylolytic activity	1.028 ± 0.094	Potato rot, Moscow region, 2019	QB073600	-
		Cellulolytic activity	1.294 ± 0.143			
		Proteolytic activity	1.020 ± 0.011			
		Pectinolytic activity	1.026 ± 0.096			
Aspergillus niger	RT598	Amylolytic activity	1.037 ± 0.052	Penza region, maize, 2023	QB073599	-
Trichoderma harzianum	Th2 (Th2RI99)	Cellulolytic activity	1.020 ± 0.115	Pesticide Rizoderma (Rizobacter Argentina S.A., Argentina)	QB073601	Barrera V.A. et al., doi: 10.1080/00275514.2021.1947641.
Bacillus amyloliquefaciens	MBI 600	Proteolytic activity	4.685 ± 0.925	Pesticide Serifel 9.9 WP (BASF Hellas S.A.), 2020	CP033205.1; published MBI 600 genome, reference record	Samaras A., et al. doi: 10.3389/fmicb.2020.600393.
Fusarium graminearum	PH-1 = NRRL 31084 = CBS 123657	Pectinolytic activity	1.078 ± 0.150	*Zea mays*, Michigan, USA	GCF_000240135.3; reference PH-1 genome assembly, RefSeq	Cuomo C.A., doi: 10.1126/science.1143708.

Note. REA values are presented as the mean ± standard deviation (n = 3). REA, relative enzymatic activity. Positive-control cultures were used to verify the performance of the corresponding substrate agar assays. Public genomic records for MBI 600 (CP033205.1) and PH-1 (GCF_000240135.3) are provided as references and are not results of sequencing the control cultures in the present study. REA values for the control cultures are presented descriptively and were not included in the one-way analysis of variance or multiple comparisons performed for the studied *Epicoccum* isolates.

Supplementary Table S10. Relative extracellular enzymatic activity of the studied *Epicoccum* isolates in substrate agar assays.

Isolate	Species	Amylolytic REA		Pectinolytic REA		Cellulolytic REA		Proteolytic REA	
		Mean value ± SD	Duncan multiple range test (grouping letters)	Mean value ± SD	Duncan multiple range test (grouping letters)	Mean value ± SD	Duncan multiple range test (grouping letters)	Mean value ± SD	Duncan multiple range test (grouping letters)
164H	*E. oryzae*	1.0490 ± 0.1952	cd	1.0211 ± 0.1228	a	0.9679 ± 0.1567	a	0.0000 ± 0.0000	-
197H	*E. mezzettii*	1.2685 ± 0.1354	bc	1.0275 ± 0.0562	a	0.9811 ± 0.1688	a	0.0000 ± 0.0000	-
250H	*E. mezzettii*	1.4985 ± 0.1355	b	0.0000 ± 0.0000	b	1.0000 ± 0.0562	a	0.0000 ± 0.0000	-
159H	*E. mezzettii*	1.1963 ± 0.1171	cd	1.0288 ± 0.1912	a	1.0278 ± 0.1070	a	0.0000 ± 0.0000	-
190H	*E. mezzettii*	1.8896 ± 0.1340	a	1.0346 ± 0.0777	a	0.9883 ± 0.0581	a	0.0000 ± 0.0000	-
46H	*E. mezzettii*	1.0125 ± 0.0433	cd	1.0130 ± 0.1056	a	1.0317 ± 0.1732	a	0.0000 ± 0.0000	-
193H	*E. tobaicum*	1.0161 ± 0.0671	cd	0.0000 ± 0.0000	b	1.0000 ± 0.0065	a	0.0000 ± 0.0000	-
152H	*E. tobaicum*	1.1147 ± 0.2115	cd	0.0000 ± 0.0000	b	0.0000 ± 0.0000	b	0.0000 ± 0.0000	-
288H	*E. tobaicum*	1.0599 ± 0.0870	cd	1.0303 ± 0.1754	a	1.0967 ± 0.1615	a	0.0000 ± 0.0000	-
17H	*E. tobaicum*	1.0410 ± 0.1788	cd	1.0000 ± 0.1620	a	1.0000 ± 0.0124	a	0.0000 ± 0.0000	-
124H	*E. tobaicum*	1.0704 ± 0.1296	cd	0.9772 ± 0.1727	a	1.0339 ± 0.1738	a	0.0000 ± 0.0000	-
143H	*E. tobaicum*	1.1131 ± 0.0197	cd	1.0310 ± 0.1116	a	1.0000 ± 0.1180	a	0.0000 ± 0.0000	-
200H	*E. tobaicum*	0.9807 ± 0.0791	d	1.0699 ± 0.0915	a	1.0101 ± 0.1602	a	0.0000 ± 0.0000	-

Isolate	Species	Amylolytic REA		Pectinolytic REA		Cellulolytic REA		Proteolytic REA	
		Mean value ± SD	Duncan multiple range test (grouping letters)	Mean value ± SD	Duncan multiple range test (grouping letters)	Mean value ± SD	Duncan multiple range test (grouping letters)	Mean value ± SD	Duncan multiple range test (grouping letters)
120H	*E. tobaicum*	1.0556 ± 0.1084	cd	1.0636 ± 0.0630	a	1.0000 ± 0.1808	a	0.0000 ± 0.0000	-
133H	*E. tobaicum*	1.4899 ± 0.2447	b	1.0307 ± 0.0019	a	1.0328 ± 0.1848	a	0.0000 ± 0.0000	-

Note. Values are presented as the mean ± standard deviation (n = 3). Differences among isolates were evaluated using one-way analysis of variance (ANOVA) followed by Duncan's multiple range test. Different lowercase letters within a given enzymatic assay indicate statistically significant differences among isolates at $p < 0.05$. Proteolytic activity was not statistically compared because REA = 0 for all study isolates in all replicates. REA, relative enzymatic activity.

Supplementary Table S11. Spearman rank correlations among aggressiveness, reduction in seed germination, rate of increase in colony diameter, and extracellular enzymatic activity of *Epicoccum* isolates.

Trait 1	Trait 2	n	Spearman's ϱ	p	FDR-adjusted p (BH)
Leaf necrotic lesion diameter, mm	Reduction in seed germination, %	15	-0.1149	0.6834	0.7886
Leaf necrotic lesion diameter, mm	Mean growth rate on PDA, MEA, and OA, mm/day	15	0.4429	0.0983	0.3686
Leaf necrotic lesion diameter, mm	Amylolytic activity, REA	15	0.0643	0.8199	0.8785
Leaf necrotic lesion diameter, mm	Cellulolytic activity, REA	15	0.5710	0.0262	0.3686
Leaf necrotic lesion diameter, mm	Pectinolytic activity, REA	15	0.2437	0.3814	0.7886
Reduction in seed germination, %	Mean rate of increase in colony diameter across the three media, mm/day	15	0.1795	0.5220	0.7886
Reduction in seed germination, %	Amylolytic activity, REA	15	0.2514	0.3662	0.7886
Reduction in seed germination, %	Cellulolytic activity, REA	15	-0.1554	0.5802	0.7886
Reduction in seed germination, %	Pectinolytic activity, REA	15	-0.4541	0.0891	0.3686
Mean rate of increase in colony diameter across the three media, mm/day	Amylolytic activity, REA	15	-0.2357	0.3977	0.7886
Mean rate of increase in colony diameter across the three media, mm/day	Cellulolytic activity, REA	15	0.4510	0.0916	0.3686
Mean rate of increase in colony diameter across the three media, mm/day	Pectinolytic activity, REA	15	0.1183	0.6746	0.7886
Amylolytic activity, REA	Cellulolytic activity, REA	15	-0.1855	0.5081	0.7886
Amylolytic activity, REA	Pectinolytic activity, REA	15	0.0108	0.9697	0.9697
Cellulolytic activity, REA	Pectinolytic activity, REA	15	0.1716	0.5410	0.7886

Note. Correlations were calculated at the level of individual isolates using one mean value of each trait for each isolate (n = 15). Spearman rank correlation coefficients (ϱ), raw p-values, and p-values adjusted using the Benjamini-Hochberg procedure to control the false discovery rate (FDR) across 15 pairwise comparisons are presented. Differences were considered statistically significant at an adjusted $p < 0.05$. Proteolytic activity was not included in the correlation analysis because this trait showed no variability (REA = 0 for all study isolates).

Supplementary Table S12. *In vitro* sensitivity of the studied *Epicoccum* isolates to azoxystrobin, boscalid, fludioxonil, and tebuconazole.

Isolate	Species	Azoxystrobin EC_{50} ± SD, μg/mL	Boscalid EC_{50}		Fludioxonil EC_{50}		Tebuconazole EC_{50}	
			Mean value ± SD, μg/mL	Duncan multiple range test (grouping letters)	Mean value ± SD, μg/mL	Duncan multiple range test (grouping letters)	Mean value ± SD, μg/mL	Duncan multiple range test (grouping letters)
164H	*E. oryzae*	>1000	0.2411 ± 0.0042	d	0.1028 ± 0.0016	i	12.396 ± 3.207	c
197H	*E. mezzettii*	>1000	0.1835 ± 0.0017	e	0.2132 ± 0.0073	cd	41.132 ± 1.035	ab
250H	*E. mezzettii*	>1000	0.2785 ± 0.0056	c	0.1950 ± 0.0114	de	49.542 ± 2.367	a
159H	*E. mezzettii*	>1000	0.2712 ± 0.0214	c	0.1463 ± 0.0021	g	11.495 ± 1.059	c
190H	*E. mezzettii*	>1000	0.3383 ± 0.0244	b	0.1199 ± 0.0005	h	9.613 ± 0.594	cd
46H	*E. mezzettii*	>1000	0.3837 ± 0.0026	a	0.2306 ± 0.0248	bc	35.827 ± 1.963	b
193H	*E. tobaicum*	>1000	0.0448 ± 0.0016	k	0.1832 ± 0.0031	ef	5.678 ± 1.281	fg
152H	*E. tobaicum*	0.787 ± 0.212	0.0857 ± 0.0020	h	0.0760 ± 0.0026	j	5.009 ± 0.598	gh
288H	*E. tobaicum*	>1000	0.1458 ± 0.0070	f	0.2262 ± 0.0116	bc	7.099 ± 1.280	ef
17H	*E. tobaicum*	>1000	0.1727 ± 0.0211	e	0.2736 ± 0.0381	a	3.735 ± 0.366	i
124H	*E. tobaicum*	>1000	0.0683 ± 0.0019	i	0.2442 ± 0.0094	b	7.355 ± 1.083	e
143H	*E. tobaicum*	>1000	0.0612 ± 0.0020	j	0.2180 ± 0.0073	bcd	8.828 ± 0.319	de
200H	*E. tobaicum*	>1000	0.1094 ± 0.0024	g	0.1693 ± 0.0064	f	4.756 ± 0.451	ghi
120H	*E. tobaicum*	>1000	0.0780 ± 0.0135	h	0.1991 ± 0.0262	de	4.199 ± 1.072	hi
133H	*E. tobaicum*	>1000	0.1171 ± 0.0023	g	0.2334 ± 0.0048	bc	10.781 ± 0.508	cd

Note. EC_{50} values are presented as the mean ± standard deviation of three independently calculated values (n = 3). For boscalid, fludioxonil, and tebuconazole, statistical analysis was performed on log10-transformed EC_{50} values using one-way analysis of variance (ANOVA) followed by Duncan's multiple range test. Different lowercase letters within a given active ingredient indicate statistically significant differences among isolates at $p < 0.05$. For azoxystrobin, a value >1000 μg/mL indicates that 50% growth inhibition was not achieved at the highest tested concentration of 1000 μg/mL; these right-censored values were not included in ANOVA or multiple comparisons. EC_{50} - concentration of active ingredient causing 50% inhibition of mycelial growth. Tebuconazole EC_{50} values were below or close to the lowest tested concentration (50 μg/mL) and were obtained by extrapolation of the fitted 4PL curves; they should be regarded as approximate.

Supplementary Table S13. Summary results of one-way analysis of variance for quantitative traits of the studied *Epicoccum* isolates

Experiment / trait	Analyzed variable	Transformation	df (between; error)	F	p	η^2
Growth rate on PDA	Rate of increase in colony diameter, mm/day	none	14; 30	101.268	<0.001	0.979
Growth rate on MEA	Rate of increase in colony diameter, mm/day	none	14; 30	180.632	<0.001	0.988
Growth rate on OA	Rate of increase in colony diameter, mm/day	none	14; 30	187.808	<0.001	0.989
Leaf assay, mycelial plugs	Necrotic lesion diameter, mm	none	15; 114	15.090	<0.001	0.665
Leaf assay, wound-assisted conidial inoculation	Proportion of necrotic leaflet area, %	arcsin√(p/100)	9; 50	21.335	<0.001	0.793
Seed assay	Germination energy, %	arcsin√(p/100)	15; 32	38.747	<0.001	0.948
Seed assay	Final germination, %	arcsin√(p/100)	15; 32	52.105	<0.001	0.961
Seed assay	Visible seed surface colonization, %	arcsin√(p/100)	15; 32	663.995	<0.001	0.997
Amylolytic activity	REA	none	14; 30	9.783	<0.001	0.820
Pectinolytic activity	REA	none	14; 30	44.456	<0.001	0.954
Cellulolytic activity	REA	none	14; 30	11.802	<0.001	0.846
Boscalid	EC_{50}, µg/mL	log10(EC_{50})	14; 30	320.121	<0.001	0.993
Fludioxonil	EC_{50}, µg/mL	log10(EC_{50})	14; 30	93.514	<0.001	0.978
Tebuconazole	EC_{50}, µg/mL	log10(EC_{50})	14; 30	102.908	<0.001	0.980

Note. The arcsin√(p/100) transformation was used for percentage variables in the seed experiment, and $\log_{10}$ transformation was used for EC_{50}. Azoxystrobin was not included in ANOVA because most values were right-censored (>1000 µg/mL). Proteolytic activity was not analyzed statistically because of the absence of variability. In the conidial assay, the individual plant was considered the experimental unit; for each of the nine isolates and the negative control, six values were analyzed, obtained by assessing one leaflet from each plant. Effect sizes were calculated as η^2 = (F × df_between)/(F × df_between + df_error). In the mycelial-plug leaf assay the design was slightly unbalanced (n = 8 per treatment, except isolates 133H and 143H with n = 9), giving 130 observations and 114 error degrees of freedom. Assessment of the ANOVA assumptions for the seed experiment is reported in Section 4.9.
